# Early occupational intervention for people with low back pain in physically demanding jobs: A randomized clinical trial

**DOI:** 10.1371/journal.pmed.1002898

**Published:** 2019-08-16

**Authors:** Bjarke Brandt Hansen, Lilli Kirkeskov, Luise Moelenberg Begtrup, Mikael Boesen, Henning Bliddal, Robin Christensen, Ditte Lundsgaard Andreasen, Lars Erik Kristensen, Esben Meulengracht Flachs, Ann Isabel Kryger

**Affiliations:** 1 Parker Institute, Copenhagen University Hospital, Bispebjerg and Frederiksberg, Denmark; 2 Department of Occupational and Environmental Medicine, Copenhagen University Hospital, Bispebjerg and Frederiksberg, Denmark; 3 Center of Social Medicine, Copenhagen University Hospital, Bispebjerg and Frederiksberg, Denmark; 4 Department of Radiology, Copenhagen University Hospital, Bispebjerg and Frederiksberg, Denmark; 5 Research Unit of Rheumatology, Department of Clinical Research, University of Southern Denmark, Odense University Hospital, Odense, Denmark; Cabrini Hospital and Monash University, AUSTRALIA

## Abstract

**Background:**

Occupational medicine seeks to reduce sick leave; however, evidence for an add-on effect to usual care is sparse. The objective of the GOBACK trial was to test whether people with low back pain (LBP) in physically demanding jobs and at risk of sick leave gain additional benefit from a 3-month complex intervention that involves occupational medicine consultations, a work-related evaluation and workplace intervention plan, an optional workplace visit, and a physical activity program, over a single hospital consultation and an MRI.

**Methods and findings:**

We enrolled people from the capital region of Denmark to an open-label, parallel-group randomized controlled trial with a superiority design from March 2014 through December 2015. In a hospital setting 305 participants (99 women) with LBP and in physically demanding jobs were randomized to occupational intervention (*n =* 153) or no additional intervention (control group; *n =* 152) added to a single hospital consultation giving a thorough explanation of the pain (i.e., clinical examination and MRI) and instructions to stay active and continue working. Primary outcome was accumulated sick leave days due to LBP during 6 months. Secondary outcomes were changes in neuropathic pain (painDETECT questionnaire [PDQ]), pain 0–10 numerical rating scale (NRS), Fear-Avoidance Beliefs Questionnaire (FABQ), Roland–Morris Disability Questionnaire (RMDQ), Short Form Health Survey (SF-36) for physical and mental health-related quality of life (HRQoL), and self-assessed ability to continue working (range 0–10). An intention-to-treat analysis of sick leave at 6 months showed no significant difference between groups (mean difference in days suggestively in favor of no additional intervention: 3.50 [95% CI –5.08 to 12.07], *P =* 0.42). Both groups showed significant improvements in average pain score (NRS), disability (RMDQ), fear-avoidance beliefs about physical activities and work (FABQ), and physical HRQoL (SF-36 physical component summary); there were no significant differences between the groups in any secondary outcome. There was no statistically significant improvement in neuropathic pain (PDQ score), mental HRQoL (SF-36 mental component summary), and self-assessed ability to stay in job. Four participants could not complete the MRI or the intervention due to a claustrophobic attack or accentuated back pain. Workplace visits may be an important element in the occupational intervention, although not always needed. A per-protocol analysis that included the 40 participants in the intervention arm who received a workplace visit as part of the additional occupational intervention did not show an add-on benefit in terms of sick leave (available cases after 6 months, mean difference: –0.43 days [95% CI –12.8 to 11.94], *P =* 0.945). The main limitations were the small number of sick leave days taken and that the comprehensive use of MRI may limit generalization of the findings to other settings, for example, general practice.

**Conclusions:**

When given a single hospital consultation and MRI, people in physically demanding jobs at risk of sick leave due to LBP did not benefit from a complex additional occupational intervention. Occupational interventions aimed at limiting biopsychological obstacles (e.g., fear-avoidance beliefs and behaviors), barriers in the workplace, and system barriers seem essential to reduce sick leave in patients with LBP. This study indicates that these obstacles and barriers may be addressed by thorough usual care.

**Trial registration:**

Clinical Trials.gov: NCT02015572

## Abstract 2

In a randomized trial, Bjarke Brandt Hansen and colleagues study a complex occupational intervention for patients with low back pain.

## Author summary

**Why was this study done?:**

- Individuals with low back pain (LBP), especially those in physically demanding jobs, are at risk of taking sick leave.
- The resultant loss of productivity adds to the already considerable socioeconomic burden of LBP.
- While occupational medicine interventions can help to reduce sick leave, there is limited evidence available on the effect of such interventions in people at risk of sick leave due to LBP who receive a thorough single hospital consultation.

**What did the researchers do and find?:**

- This parallel-group randomized clinical trial compared an early occupational intervention added to usual care with usual care alone in patients with LBP and physically demanding jobs; of note, usual care included a clinical examination and magnetic resonance imaging scan to enable a thorough explanation of the pain, and recommendations to stay active in work.
- The trial showed no significant difference between the treatment groups in terms of accumulated sick leave during a 6-month period, although both groups did show improvements from baseline in pain, fear-avoidance beliefs, physical quality of life, and disability at 6 months.

**What do these findings mean?:**

- This study indicates that LBP interventions that include an explanation of the pain might be sufficient to limit sick leave in patients with LBP and physically demanding jobs; an approach that could be integrated into usual care and an early occupational intervention would not necessarily have to be carried out by a specialist in occupational medicine.

## Introduction

The lifetime prevalence of low back pain (LBP) is about 70% [1]. In the US alone, an estimated $87 billion is spent annually on healthcare for individuals with back pain, which has been one of the fastest growing healthcare expenses [2]. Sick leave and productivity loss add to this considerable socioeconomic burden [3]. Despite considerable resources being used to prevent LBP, the incidence curve has still not declined, and this has led to greater attention on tertiary prevention [4,5]. Occupational attachment is associated with physical and mental well-being [6]. Therefore, attachment to the labor market is recommended in the management of patients who have developed LBP [7,8], and cognitive behavioral therapy focusing on biopsychosocial aspects (e.g., fear-avoidance behavior) has proven effective [5,9,10]. Combining workplace interventions with physical exercise seems to reduce LBP disability and sick leave among workers with musculoskeletal disorders [7,11]. Recent studies of interventions for chronic LBP focusing on occupational attachment found not only a decrease in disability but also a better cost-effectiveness when compared with usual care in a primary healthcare setting [10,11]. A similar effect has been seen on sick leave in a secondary healthcare setting [12]. Most guidelines for LBP care advocate the use of reassurance, analgesics, and recommendations to stay active and continue working [5]. A thorough explanation of the pain has been successful in altering fear-avoidance behavior and seems to reduce sick leave in patients with LBP [13,14]. Occupational intervention is usually given as an add-on to usual care. Therefore, the GOBACK trial was conducted to test whether individuals with LBP in physically demanding jobs at risk of sick leave gain further benefit when adding a 3-month complex early occupational intervention to a single hospital consultation, when this consultation includes a thorough explanation of the pain (i.e., clinical examination and MRI) and recommendations to stay active and continue working.

## Methods

### Design setting and participants

The GOBACK trial protocol has been published elsewhere [15], and the prespecified statistical analysis plan (S1 Text) and the Consolidated Standards of Reporting Trials (CONSORT) checklist (S2 Text) are provided. The GOBACK trial was a 6-month, pragmatic, single-center, open-label, parallel-group randomized clinical trial with a superiority design. It was approved by the local ethics committee (H-3-2013-161) and by the Danish Data Protection Agency (DPA: 2014-41-2673). The study was done in a hospital setting in Frederiksberg, Denmark.

The participants were recruited from an advertisement in a newspaper or referred from general practices or the study hospital’s Department of Rheumatology. Potential participants were contacted by telephone and screened for eligibility. After providing written informed consent, participants were scheduled for hospital consultation including the baseline assessment, followed by random assignment to the additional occupational intervention (intervention group) or no add-on (control group) and a 6-month follow-up assessment. An investigator (RC) not related to the intervention or outcome assessments performed the allocation.

Eligible participants were aged 18 to 65 years with a current episode of 2–4 weeks of LBP and a self-reported physically demanding job, who—independent of sick leave status and previous history of LBP—expressed concerns about their ability to continue working (minimum 30 hours/week). A physically demanding job was defined by the participants “agreeing” or “strongly agreeing” on the question whether their job was physically demanding. Exclusion criteria were pregnancy, severe somatic or psychiatric disease, cancer or metastatic disease, treatment or referral to outside providers (e.g., surgery), or contraindications for having magnetic resonance imaging (MRI).

### Outcome assessments

The clinical examination and an MRI scan of the lumbar spine were included in the baseline assessment, and questionnaires were answered using a validated touchscreen without involvement of the assessors [16]. To further characterize workload, all participants’ job titles were classified using the Danish version of the International Standard Classification of Occupations (DISCO-88) [17]. A junior medical physician (BBH) or a specialist in rheumatology (LEK) performed a clinical examination and reviewed clinically relevant health-related answers, noting if further examinations were necessary. A radiologist evaluated the MRI scans, and the participants were informed of the findings by telephone 2–3 weeks after the baseline assessment. The information was presented in a way to reduce fear of a severe condition causing the back pain in the participants. The physicians who performed the clinical examinations were not allowed to participate in the occupational intervention but were allowed to take action if the physical examination revealed conditions that needed further clinical intervention. In addition, the 6-month follow-up assessment was blinded for allocation by instructing the participants not to mention their allocation.

The primary outcome was accumulated sick leave, in full days, due to LBP during the 6 months from baseline. The participants reported their sick leave weekly in a paper-based diary, and they received a weekly text message with a reminder to fill in the diary. Sick leave due to reasons other than LBP was not included in this study. Secondary outcomes, assessed at baseline and after 6 months, included a screening questionnaire to identify neuropathic components of participants’ back pain (painDETECT questionnaire [PDQ]) [18], LBP severity on a 0–10 numeric rating scale (NRS) [19], and back-pain-related disability by the 24-item Roland–Morris Disability Questionnaire (RMDQ) modified to 23 items [20] and converted to a 0–100 scale [21]. Further secondary outcomes included the Fear-Avoidance Beliefs Questionnaire (FABQ) for physical activity and work [22]; the Short Form Health Survey (SF-36) questionnaire for physical and mental health-related quality of life (HRQoL), expressed in 2 composite scores, a physical component summary score and a mental component summary score, in which the ordinal scale scores were transformed into linear scales ranging from 0 to 100 [23]; and self-assessed ability to continue working on a 0 to 10 NRS, with higher scores indicating better ability to stay in job. Overall satisfaction with the intervention was rated on a 5-point NRS, with the anchors “not at all satisfied” = 0 and “extremely satisfied” = 4.

### Randomization

After the baseline assessment and the consultation with the medical physician, participants were randomized 1:1 to either the additional occupational intervention or no add-on. The randomization was based on a computer-generated list (random permuted block design using block sizes of 2, 4, or 6) generated by an independent statistician and administrated by sealed envelopes. The participants were stratified by age at enrollment (<40 years or ≥40 years) and sex (male/female).

### Interventions

All participants received the single hospital consultation, consisting of a clinical examination and an MRI scan to give an explanation of the pain, and recommendations to stay active and continue working. The additional occupational intervention lasted 3 months and started with an initial consultation with an occupational medicine physician, who performed a work-related evaluation and provided guidance to address biopsychosocial obstacles and fear-avoidance behavior towards work. In collaboration with the participant, a workplace intervention plan was developed, with an optional workplace visit to address ergonomic obstacles. A physical therapist guided the participant to remain as active as possible by performing a 45-minute self-administered physical activity program 3 times weekly. There was a midway consultation after 6 weeks, to ensure that the workplace intervention and training were followed and necessary adjustments to the plan could be made. Finally, there was an end consultation at 3 months with an occupational medicine physician, who evaluated the intervention and provided further guidance to the participant. The participants were contacted weekly during the first month and every second week during the following 2 months to encourage the participant to follow the intervention and training. The intervention has been described in further detail in the protocol [15].

### Safety

Independent of allocation, participants were able to contact the trial’s physicians in case of an adverse event (e.g., temporarily increased pain or neurological symptoms). All participants received usual care, and no treatment was withheld from the participants during the trial.

### Sample size and statistical analysis

The prespecified sample-size calculation found that 127 participants would be required in each group to obtain 80% power to detect a mean difference in sick leave of 6 days between the treatment groups (2-sample pooled *t* test; *P =* 0.05), assuming a standard deviation (SD) of 17 days [24]. Expecting some dropouts during the trial period (<20%), we decided to enroll 300 participants in total (≥150 participants in each group).

Baseline characteristics are presented by group. Analyses of primary and secondary outcomes were based on the intention-to-treat population and conducted by analysis of covariance, including treatment group, age, sex, and baseline value of the relevant variable as covariates. Multiple imputation was used for missing observations. To test the robustness of the analyses, we also explored the sensitivity of the overall conclusions to various limitations of the data, assumptions, and analytic approaches to data analysis. These analyses included available case analysis and non-responder imputation using a baseline observation carried forward (BOCF) approach for missing data. Furthermore, a per-protocol analysis was conducted including only participants who received a workplace visit as part of the occupational intervention. A post hoc analysis was also conducted that included only participants who reported having very physically demanding jobs. All statistical tests were 2-sided, with *P* values < 0.05 considered statistically significant, and were carried out using R 3.0.1 (http://www.R-project.org; R Foundation for Statistical Computing). Additional methodological details are outlined in our statistical analysis plan (S1 Text). This trial is registered at ClinicalTrials.gov (number NCT02015572).

## Results

### Baseline characteristics

**Fig 1:**
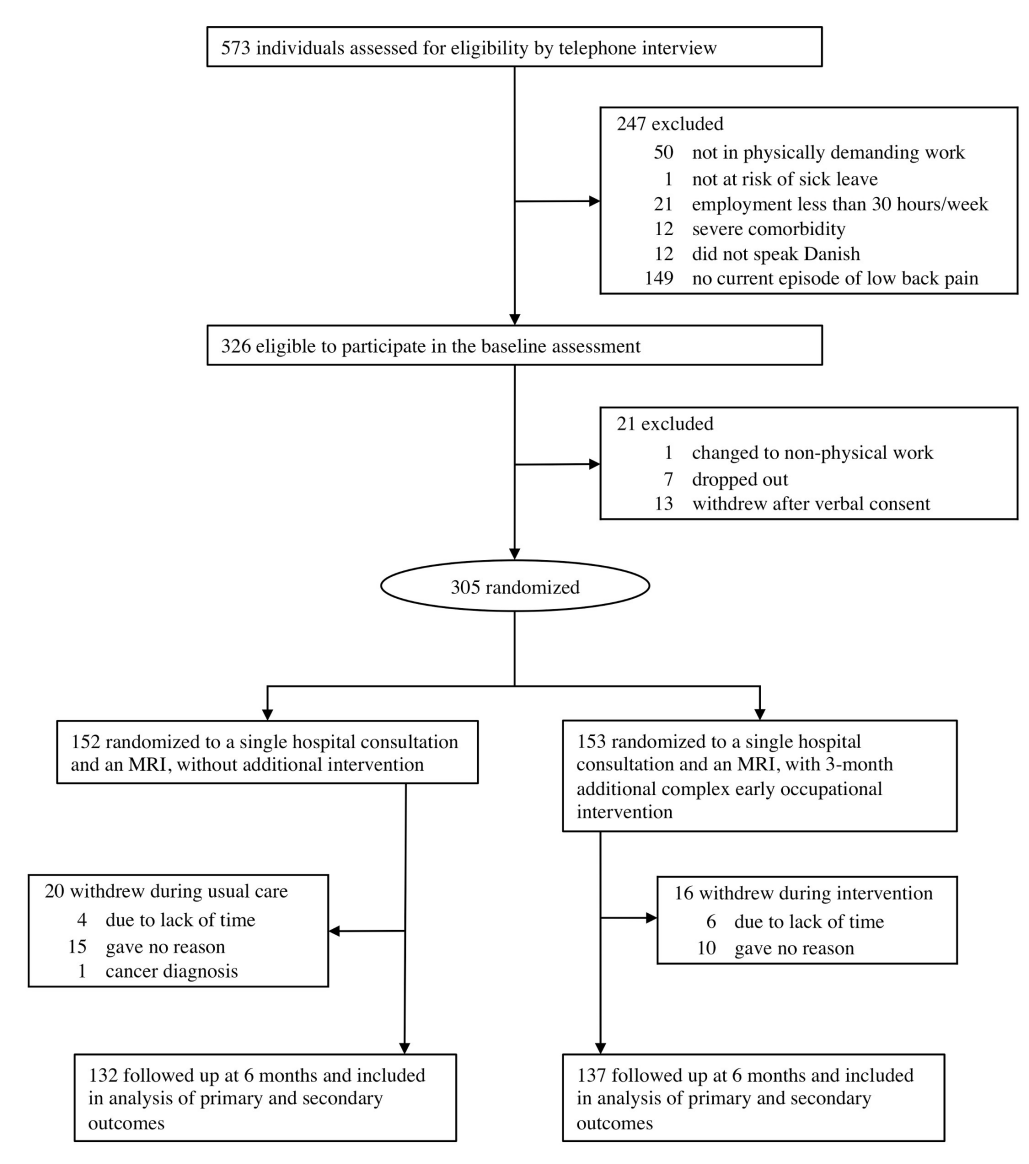
Flow of participants through the trial.

From 7 March 2014 to 17 December 2015, 573 potentially eligible participants were identified. Of those, a majority (*n =* 556) were recruited via newspaper advertisements, and 326 were assigned for baseline assessment. One participant changed to non-physical work, 7 did not attend the baseline assessment, and 13 withdrew verbal consent. Therefore, randomization assigned 153 participants to the intervention group and 152 participants to the control group (Fig 1). The mean age was 45.5 years (SD 10.3), and, as expected due to the inclusion criteria, more men than women (32.5%) were enrolled (Table 1). Overall, 226 (74.1%) participants reported more than 3 months with back pain, thus characterized as having chronic LBP at baseline [4]. Based on the DISCO-88 job title categorizations, 59.3% of the participants’ jobs were classified as manual labor; 33.8% as office work, technical, or health related (e.g., nursing); and 6.9% as administrative [17]. Mean [SD] FABQ (25.0 [7.4]) and self-assessed ability to continue working (6.24 [2.03]) indicated a high level of fear-avoidance beliefs towards work [25]. The clinical examination resulted in 15 participants being referred to additional outside providers: MRI indicated inflammatory rheumatic spine diseases in 11 participants, who were referred to a rheumatologist; 3 participants were referred to an orthopedic surgeon due to additional symptoms of hip osteoarthritis; and 1 was referred to a spine surgeon.

**Table 1:** Baseline characteristics.

Characteristic	No additional intervention (*n =* 152)	Additional occupational intervention (*n =* 153)	All participants (*n =* 305)
**Sociodemographic characteristics**			
Female sex	50 (32.9%)	49 (32.0%)	99 (32.5%)
Age, years	45.7 (10.5)	45.3 (10.1)	45.5 (10.3)
Current smoker	63 (41.5%)	49 (32.0%)	112 (36.7%)
BMI, kg/m^2^	27.2 (4.3)	26.7 (4.14)	26.9 (4.2)
>9 years education	112 (73.7%)	92 (60.1%)	204 (66.9%)
**Employment characteristics**			
Job category DISCO-88 code > 5	83 (54.6%)	98 (64.1%)	181 (59.3%)
Self-assessed ability to continue in work	6.23 (1.87)	6.24 (2.18)	6.24 (2.03)
Self-reported current physical workload			
Very demanding	75 (49.3%)	69 (45.1%)	144 (47.2%)
Demanding	73 (48.0%)	81 (52.9%)	154 (50.5%)
Medium demanding	4 (2.6%)	3 (2.0%)	7 (2.3%)
Sick leave due to LBP last year > 7 days	68 (44.7%)	69 (45.0%)	137 (44.9%)
Sick leave due to LBP last year > 1 months	17 (11.2%)	16 (10.5%)	33 (10.8%)
**Low back pain characteristics**			
Duration of LBP ≥ 3 months	110 (72.4%)	116 (75.8%)	226 (74.1%)
NRS for pain	5.5 (1.98)	5.7 (1.88)	5.6 (1.93)
LBP without sciatica/radiculopathy	84 (55.3%)	86 (56.2%)	170 (55.7%)
Neurologic abnormalities/deficits	23 (15.1%)	26 (17.0%)	49 (16.1%)
PDQ	11.9 (6.5)	11.3 (6.1)	11.6 (6.3)
RMDQ	51.5 (22.7)	48.3 (22.7)	49.9 (22.7)
FABQ–Physical activity	14.6 (5.0)	14.6 (5.0)	14.6 (5.0)
FABQ–Work	25.2 (7.3)	24.9 (7.6)	25.0 (7.4)
**Current medications for back pain (weekly)**			
Non-steroidal anti-inflammatory	109 (71.7%)	113 (74.3%)	222 (72.8%)
Opioid	32 (21.1%)	38 (24.8%)	70 (23.0%)
Muscle relaxer/anticonvulsant	5 (3.3%)	1 (0.7%)	6 (2.0%)
Steroid anti-inflammatory	3 (2.0%)	3 (2.0%)	6 (2.0%)
Other (e.g., acetaminophen)	134 (88.2%)	138 (90.2%)	272 (89.2%)
**Health-related quality of life**			
SF-36–PCS for physical health	37.7 (7.6)	38.1 (8.2)	37.9 (7.9)
SF-36–MCS for mental health	48.4 (10.9)	48.0 (10.7)	48.2 (10.8)
Magnetic resonance imaging*			
Herniation	77 (52.0%)	76 (51.4%)	153 (51.7%)
Spinal stenosis	25 (16.9%)	16 (10.8%)	41 (13.9%)
Inflammatory spinal disease	7 (4.8%)	5 (3.4%)	12 (4.1%)
Spondylolisthesis	17 (11.5%)	27 (18.2%)	44 (14.9%)
Non-specific spondylosis	83 (56.1%)	88 (59.5%)	171 (57.8%)
Morbus Scheuermann stigmata	7 (4.8%)	8 (5.4%)	15 (5.1%)

Data are given as *n* (%) or mean (SD). For the Danish version of the International Standard Classification of Occupations (DISCO-88), categories range from 1 to 9, with job categories over 5 indicating manual labor and/or physical work (blue-collar workers). Ability to stay in job was assessed on a 0 to 10 scale, with higher scores indicating better ability to stay in job. For the numeric rating scale (NRS) for pain, scores range from 0 to 10, with higher scores indicating more pain. For the painDETECT questionnaire (PDQ), scores range from 0 to 30, with higher scores indicating more neuropathic pain. For the Roland–Morris Disability Questionnaire (RMDQ), scores range from 0 to 100 after converting from a 24-item scale to a 23-item scale, with higher scores indicating greater disability. For the Fear-Avoidance Beliefs Questionnaire (FABQ) subscale for physical activity, the scores range from 0 to 24, with higher scores indicating greater fear-avoidance beliefs towards physical activities, and for the subscale for work, the scores range from 0 to 42, with higher scores indicating greater fear-avoidance beliefs towards work. For the Short Form Health Survey (SF-36) physical component summary (PCS), the scores range from 0 to 100, with higher scores indicating better physical function, and for the mental composite summary (MCS), the scores range from 0 to 100, with higher scores indicating better mental health.

*Five in the intervention group and 4 in the control group did attend the magnetic resonance imaging (MRI) examination or had their MRI examination interrupted due to claustrophobic attack.

LBP, low back pain.

### Adherence to the interventions

Thirty-six participants (11.8% of the total participants; 16 in the intervention group and 20 in the control group) dropped out during the trial; 25 gave no reason, 10 cited lack of time due to work responsibilities, and 1 was referred for treatment for a gynecological cancer (Fig 1). All the participants allocated to the additional intervention (the intervention group) attended the initial consultation and the midway consultation. Workplace visits were considered relevant for 55 (35.9%) participants. However, these visits took place in only 40 cases (27.2%), as 15 participants were concerned that the visit would increase the risk of being dismissed from their job. The physical therapist reported that 127 (83.0%) participants adhered to the physical activity plan. Reasons for non-adherence were lack of time (*n =* 15), back pain (*n =* 5), or other conditions that needed attention (*n =* 6). In total, 137 participants (89.5%) attended the end consultation, and 51.8% were very satisfied with the intervention (for more details, see S1 Table).

### Primary and secondary outcomes

More than 70% of the participants in each group reported fewer than 7 days of sick leave due to back pain during the 6 months. The intention-to-treat analysis with imputations for non-responders of sick leave at 6 months did not show a significant add-on benefit of the occupational intervention (mean difference between groups in days: 3.50 [95% CI –5.08 to 12.07], *P =* 0.42; Table 2). This result was also found in an additional analysis based on the available cases at 6 months (mean difference between groups in days without imputation applied: 0.12 [95% CI –7.90 to 8.15], *P =* 0.97; for more details, see S2 Table).

**Table 2:** Changes in primary and secondary outcomes for a single hospital consultation with or without an additional occupational intervention in individuals in physically demanding jobs and at risk of sick leave.

Outcome	Change from baseline to 6-month follow-up		Comparison	
	No additional intervention (*n =* 152)	Additional occupational intervention (*n =* 153)	Mean difference between groups (95% CI)	*P* value
**Primary outcome**				
Cumulative self-reported sick leave during 6 months from baseline, mean days (SD)	15.49 (15.55)	18.54 (18.68)	3.50 (–5.08 to 12.07)	0.422
Cumulative self-reported sick leave less than 7 days, *n* (%)*	93 (70.5%)	97 (70.8%)	OR = 0.94 (0.53 to 1.66)	0.822
**Secondary outcomes**				
PDQ score for neuropathic pain	−2.30 (−4.85 to 0.25)	−1.31 (−3.35 to 0.73)	0.90 (−0.25 to 2.05)	0.125
NRS pain intensity	−1.50 (−2.36 to −0.65)	−1.84 (−2.79 to −0.89)	0.04 (−0.39 to 0.47)	0.854
RMDQ score for disability	−9.79 (−19.39 to −0.19)	−14.61 (−23.15 to −6.07)	−1.54 (−6.68 to 2.99)	0.504
FABQ score for physical activity	−2.10 (−4.40 to −0.20)	−4.25 (−6.21 to −2.26)	−0.54 (−1.77 to 0.58)	0.382
FABQ score for work	−3.77 (−6.80 to −0.74)	−3.54 (−6.87 to −0.21)	−0.23 (−1.91 to 1.46)	0.772
SF-36 physical component summary	4.41 (0.44 to 8.39)	7.54 (4.32 to 10.74)	0.20 (−1.65 to 2.04)	0.834
SF-36 mental component summary	−3.05 (−8.37 to 2.28)	3.84 (−0.64 to 8.32)	0.18 (−2.31 to 2.66)	0.890
Self-assessed ability to continue in work	0.40 (−0.59 to 1.39)	0.73 (−0.05 to 1.50)	0.06 (−0.38 to 0.50)	0.784

Data for change from baseline to 6-moth follow-up are expressed as the difference in means with 95% confidence intervals, unless otherwise indicated. The primary outcome is given as the number of days with sick leave in the 6 months after baseline. Secondary outcomes are given as mean change in the 6 months from baseline, and the comparison is given as the mean difference between groups in change from baseline. painDETECT questionnaire (PDQ) is a 0–30 scale (higher scores indicate a greater neuropathic components); numeric rating scale (NRS) is a 0–10 scale (higher scores indicate greater pain intensity); 24-item Roland–Morris Disability Questionnaire (RMDQ) is converted to a 0–100 score (higher scores indicate greater disability); Fear-Avoidance Beliefs Questionnaire (FABQ) is a 0–24 scale for physical activity (higher scores indicate greater fear-avoidance beliefs) and 0–42 scale for work (higher scores indicate greater fear-avoidance beliefs); Short Form Health Survey (SF-36) is a 0–100 scale for physical component summary (higher scores indicate higher physical function) and 0–100 scale for mental component summary (higher scores indicate higher mental health); ability to continue in work is assessed on a 0–10 scale (higher scores indicate better ability).

*No imputation applied. The comparison for this outcome is odds ratio (OR) instead of mean difference.

Both groups showed improvements in average pain score (NRS), disability (RMDQ), fear-avoidance beliefs for physical activities and work (FABQ), and physical HRQoL (SF-36 physical component summary);no statistically significant difference was found between the groups. There was no statistically significant improvement in neuropathic pain (PDQ score), mental HRQoL (SF-36 mental component summary), and self-assessed ability to stay in job in either group, and no difference between the groups. This result was also found with an alternative imputation technique (baseline observation carried forward) and in analyses including available cases (for more details, see S3 Table).

### Sensitivity analyses

We conducted additional analyses to explore whether workplace visits may be an important element in the occupational intervention and whether the intervention may be beneficial for subgroups. A per-protocol analysis that included the 40 participants who received a workplace visit as part of the additional occupational intervention was conducted. This did not show an add-on benefit in terms of sick leave (available cases after 6 months, mean difference between groups: –0.43 days [95% CI –12.8 to 11.94], *P =* 0.945; Table 3). A post hoc analysis was performed including only participants who reported their job to be very physically demanding. The analysis included 69 participants receiving the additional occupational intervention and 75 participants receiving no add-on. Again, there was no statistically significant benefit in terms of sick leave from the additional intervention (available cases after 6 months, mean difference between groups: –1.84 days [95% CI –13.48 to 9.79], *P =* 0.754; Table 4). The baseline characteristics of the 40 participants who received a workplace visit as part of the additional occupational intervention and the 144 participants who reported their job to be very physically demanding can be seen in S4 Table.

**Table 3:** Per-protocol analysis including participants who received a workplace visit as part the occupational intervention (crude estimates).

Outcome	Change from baseline to 6-month follow-up		Comparison	
	No additional intervention (*n =* 152)	Additional occupational intervention (*n =* 40)	Mean difference between groups (95% CI)	*P* value
**Primary outcome**				
Cumulative self-reported sick leave during 6 months from baseline, mean days (SD)	15.22 (37.29)	17.72 (32.33)	−0.43 (−12.8 to 11.94)	0.945
Cumulative self-reported sick leave less than 7 days, *n* (%)*	93 (70.5%)	25 (67.6%)	OR = 0.11 (0.45 to 2.60)	0.811
**Secondary outcomes**				
PDQ score for neuropathic pain	−2.43 (−3.95 to −0.91)	−0.65 (−3.55 to 2.25)	1.90 (0.04 to 3.77)	0.046
NRS pain intensity	−1.06 (−1.53 to −0.59)	−0.97 (−1.89 to −0.05)	0.21 (−0.46 to 0.89)	0.531
RMDQ score for disability	−11.50 (− 16.97 to −6.02)	−13.40 (−23.45 to −3.34)	−2.9 (−8.9 to 4.72)	0.545
FABQ score for physical activity	−2.24 (−3.47 to −1.02)	−2.14 (−4.60 to 0.33)	0.26 (−1.51 to 2.03)	0.772
FABQ score for work	−2.74 (−4.76 to −0.72)	−2.89 (−5.73 to 0.95)	−0.17 (−2.86 to 2.51)	0.900
SF-36 physical component summary	4.58 (2.60 to 6.55)	4.99 (1.20 to 8.78)	−0.50 (−3.44 to 2.44)	0.739
SF-36 mental component summary	2.00 (−1.61 to 4.60)	1.27 (−3.81 to 6.36)	−1.12 (−4.58 to 2.33)	0.522
Self-assessed ability to continue in work	0.67 (0.22 to 1.11)	0.67 (−0.15 to 1.66)	0.03 (−0.57 to 0.63)	0.929

Data are expressed as difference in means with 95% confidence intervals, unless otherwise indicated. The primary outcome is given as the number of days with sick leave in the 6 months after baseline. Secondary outcomes are given as mean change in the 6 months from baseline, and the comparison is given as the mean difference between groups in change from baseline. painDETECT questionnaire (PDQ) is a 0–30 scale (higher scores indicate a greater neuropathic components); numeric rating scale (NRS) is a 0–10 scale (higher scores indicate greater pain intensity); 24-item Roland–Morris Disability Questionnaire (RMDQ) is converted to a 0–100 score (higher scores indicate greater disability); Fear-Avoidance Beliefs Questionnaire (FABQ) is a 0–24 scale for physical activity (higher scores indicate greater fear-avoidance beliefs) and 0–42 scale for work (higher scores indicate greater fear-avoidance beliefs); Short Form Health Survey (SF-36) is a 0–100 scale for physical component summary (higher scores indicate higher physical function) and 0–100 scale for mental component summary (higher scores indicate higher mental health); ability to continue in work is assessed on a 0–10 scale (higher scores indicate better ability).

*The comparison for this outcome is odds ratio (OR) instead of mean difference.

**Table 4:** Post-hoc analysis including participants who at baseline assessment reported their job to be very physically demanding (crude estimates).

Outcome	Change from baseline to 6-month follow-up		Comparison	
	No additional intervention (*n =* 75)	Additional occupational intervention (*n =* 69)	Mean difference between groups (95% CI)	*P* value
**Primary outcome**				
Cumulative self-reported sick leave during 6 months from baseline, mean days (SD)	17.39 (39.33)	20.77 (35.87)	−1.84 (−13.48 to 9.79)	0.754
Cumulative self-reported sick leave less than 7 days, *n* (%)	41 (65.1%)	34 (56.7%)	OR = 1.28 (0.55 to 2.99)	0.561
**Secondary outcomes**				
PDQ score for neuropathic pain	−1.86 (−4.29 to 0.57)	−0.80 (−3.25 to 1.65)	1.40 (−0.45 to 3.26)	0.136
NRS pain intensity	−1.10 (−1.80 to −0.39)	−1.43 (−2.18 to −0.69)	−0.06 (−0.74 to 0.62)	0.864
RMDQ score for disability	−9.97 (−17.78 to −0.17)	−9.86 (−17.99 to −1.72)	−0.34 (−7.51 to 6.83)	0.925
FABQ score for physical activity	−2.84 (−4.54 to −1.14)	−3.88 (−5.78 to −1.99)	−0.50 (−2.30 to 1.30)	0.584
FABQ score for work	−2.41 (−5.28 to 0.45)	−3.62 (−6.54 to −0.70)	−0.95 (−3.38 to 1.47)	0.438
SF-36 physical component summary	4.65 (1.70 to 7.59)	4.25 (1.23 to 7.25)	−1.03 (−3.81 to 1.74)	0.463
SF-36 mental component summary	1.01 (−2.80 to 4.63)	1.58 (−2.57 to 5.74)	−0.53 (−4.27 to 3.20)	0.778
Self-assessed ability to continue in work	0.70 (0.03 to 1.36)	0.98 (0.16 to 1.81)	0.07 (−0.51 to 0.65)	0.812

Data are expressed as difference in means with 95% confidence intervals, unless otherwise indicated. The primary outcome is given as the number of days with sick leave in the 6 months after baseline. Secondary outcomes are given as mean change in the 6 months from baseline, and the comparison is given as the mean difference between groups in change from baseline. painDETECT questionnaire (PDQ) is a 0–30 scale (higher scores indicate a greater neuropathic components); numeric rating scale (NRS) is a 0–10 scale (higher scores indicate greater pain intensity); 24-item Roland–Morris Disability Questionnaire (RMDQ) is converted to a 0–100 score (higher scores indicate greater disability); Fear-Avoidance Beliefs Questionnaire (FABQ) is a 0–24 scale for physical activity (higher scores indicate greater fear-avoidance beliefs) and 0–42 scale for work (higher scores indicate greater fear-avoidance beliefs); Short Form Health Survey (SF-36) is a 0–100 scale for physical component summary (higher scores indicate higher physical function) and 0–100 scale for mental component summary (higher scores indicate higher mental health); ability to continue in work is assessed on a 0–10 scale (higher scores indicate better ability).

*No imputation applied. The comparison for this outcome is odds ratio (OR) instead of mean difference.

### Harms

Two participants had a claustrophobic attack, and 1 participant had accentuated back pain during the MRI examination. One participant in the intervention group reported worsening thoracic back pain during the intervention; however, additional intervention was not needed.

## Discussion

The objective of the GOBACK trial was to evaluate whether a 3-month complex early occupational intervention, given as an add-on to a single hospital consultation, decreases sick leave among patients with LBP at risk of taking sick leave during a 6-month period. Improvements from baseline to 6 months were observed in pain, fear-avoidance beliefs, physical HRQoL, and disability in both groups, and no statistically significant differences were found between the groups in accumulated sick leave (*P =* 0.422). A per-protocol analysis that included the 40 participants who received a workplace visit as part of the additional occupational intervention did not show an add-on benefit in terms of sick leave (*P =* 0.945). A post-hoc analysis was performed including only participants who reported their job to be very physically demanding. Still, there was no statistically significant benefit of the additional intervention in terms of sick leave (*P =* 0.754). These findings indicate that LBP interventions comprising an explanation of the pain based on a clinical examination and MRI, combined with instructions to stay active and continue working, might be sufficient to keep patients with physically demanding jobs and at risk of sick leave due to LBP out of sick leave.

The findings in this study add to beliefs that an explanation for back pain given by a medical physician may alter fear-avoidance beliefs and behaviors and thereby increase the odds for work participation in patients with LBP [13,14]. Interventions with a focus on limiting biopsychological obstacles (e.g., fear-avoidance beliefs and behaviors), barriers in the workplace, and system barriers seem essential to reduce sick leave in patients with LBP; these are all fundamental elements of occupational interventions [7,8,22]. However, this trial indicates that these elements may be integrated into usual care and do not necessarily have to be carried out by a specialist in occupational medicine, who can focus on sick-listed patients or primary prevention.

The findings of this trial contrast with those of previous studies showing that occupational intervention for patients with LBP seems effective for reducing both short-term (i.e., 3 months) and long-term (i.e., 12 months) sick leave compared with treatment in general practice [12,26]. In the current trial, the occupational intervention was designed to reflect normal clinic practice, and, therefore, the intervention was given as an add-on to a single hospital consultation. It is well established that most patients with acute LBP recover reasonably quickly [27]. There is therefore a substantial risk that the lack of a between-group difference in this study can be explained by the self-limiting nature of this condition. This possibility may also be supported by the small amount of sick leave in both groups during the 6-month observation period. On the other hand, in this study 74.1% of participants reported more than 3 months with back pain, and were thus characterized as having chronic LBP at baseline. In patients with chronic LBP, only about 40% recover within 12 months [27], and in our study only moderate improvements in pain were observed from baseline to 6 months in both groups.

All participants were given an MRI scan of the lumbar spine and subsequently informed of its findings to personalize the explanation of the back pain and to remove fear of severe conditions. An MRI scan is not a recommended routine examination in the diagnostics of non-specific LBP [28], and in the absence of “red flag” symptoms, most guidelines advocate for several weeks of conservative care without any diagnostic imaging [28]. Studies have also indicated that liberal use of imaging in LBP may even worsen long-term outcomes in some patients [29]. In this study, MRI was used as part of the baseline assessment to exclude fear of serious diagnoses in the patients (e.g., cancer) and to highlight the benign nature of degenerative findings. It could be speculated that this “extra attention” given to all the participants explains why we did not find a benefit from the additional intervention. However, an occupational intervention should be beneficial over a single hospital consultation (with MRI), before this became a relevant add-on in the clinic.

There is strong evidence that work-related physical factors, such as manual lifting, increase the incidence of LBP [30]. By offering an early occupational intervention to individuals who reported their job to be physically demanding, we intended to include a subgroup that was likely to benefit from such an intervention [31]. Despite an average pain level of 5.6 on the 0–10 NRS and 23% of the participants using opioids on a weekly basis to manage their pain during work, surprisingly few participants (<30%) had more than 7 days of sick leave due to LBP during the 6-month observation period. To increase sensitivity, we conducted a post-hoc analysis that included only the participants who reported their job as very demanding; however, no add-on benefit in terms of sick leave was found from the additional intervention. These findings seem to support previous findings indicating that workload is not the primary risk factor for sick leave among workers with LBP [32]. Despite this, a Cochrane review found high-quality evidence to support the use of workplace interventions among workers with musculoskeletal disorders to reduce sick leave compared with usual care [7]. It is argued that a good workplace intervention should include both the worker and the employer and that this approach may be cost-effective over usual care [33]. In the current trial, workplace visits were relevant for only 55 (35.9%) participants, which adds to the previous statement that workload is only one risk factor among many for these workers. This finding is further supported by our per-protocol analysis, which included only participants (*n =* 40) who had a workplace visit as part of their occupational intervention, and in which still no benefit from the intervention was found. Furthermore, 15 participants refused to have a workplace visit, as they were concerned the visit would increase the risk of dismissal from their job.

It may be argued that personal fitness among workers may increase the ability to adapt to the physical demands at work. For workers with LBP, physical exercise seems to reduce the number of recurrences of LBP or prolong the time to recurrence, although no particular type of exercise seems superior [34]. For this reason, our intervention’s physical exercise plan was not standardized but planned individually for each participant to increase adherence. This resulted in 83% of participants following the plan to an acceptable level after 3 months, although this may not represent a long-lasting change in behavior. To address these and other questions further, follow-up and additional analyses are planned.

The strengths of this trial are the randomized clinical trial design, the early occupational intervention to reduce sick leave, the high rate of participation during the 6 months from baseline, having sick leave as the primary outcome, the blinded assessment, and the inclusion of individuals with LBP in physically demanding jobs, which is a highly relevant subgroup for an additional occupational intervention. A significant number of participants had previous history of LBP, and therefore may already have had usual care to a varying degree before enrollment. This may have limited the chances of detecting a benefit of the complex occupational intervention. A major limitation of this trial is the skewness of data and small amount of sick leave in both groups during the 6-month observation period, which increases the risk of a floor effect. The small amount of sick leave may be explained by the inclusion of non-sick-listed patients.

Another reason for the small amount of sick leave in both groups could be that an MRI scan is not a routine examination in the diagnostics of non-specific LBP [28], and, therefore, some participants with a low risk of sick leave may have attended the trial with the purpose of having an MRI scan performed. The trial’s extensive use of MRI and the use of a single consultation in a hospital may limit the generalization of the findings to other settings, for example, general practice.

## Conclusion

When given an explanation for the pain based on a clinical examination and an MRI scan, followed by instructions to stay active and continue working, workers in physically demanding jobs at risk of sick leave due to LBP do not benefit from a 3-month complex early additional occupational intervention. This indicates that occupational elements may be integrated into usual care and do not necessarily have to be carried out by a specialist in occupational medicine, who can focus on sick-listed patients or primary prevention.

## Supporting information

S1 Table Satisfaction with the intervention. (DOCX)

S2 Table Additional analysis based on the available cases. (DOCX)

S3 Table Alternative imputation technique (baseline observation carried forward). (DOCX)

S4 Table Baseline characteristics of the per-protocol and post hoc analysis. (DOCX)

S1 Text Statistical analysis plan. (PDF)

S2 Text CONSORT checklist. (DOC)

S3 Text Protocol registration (ClinicalTrials.gov). (PDF)

S1 Alternative Language Abstract Danish translation of the abstract by BBH, LK, and AIK. (PDF)

## Abbreviations

FABQ: Fear-Avoidance Beliefs Questionnaire
HRQoL: health-related quality of life
LBP: low back pain
NRS: numerical rating scale
PDQ: painDETECT questionnaire
RMDQ: Roland–Morris Disability Questionnaire
SF-36: Short Form Health Survey
