# Supplementary material for: Early occupational intervention for people with low back pain in physically demanding jobs: A randomized clinical trial
Source: PLoS Med. 2019 Aug 16;16(8):e1002898. doi: 10.1371/journal.pmed.1002898 (PMC6697316; doi:10.1371/journal.pmed.1002898)
Supplement: S1 Table — (DOCX) [file pmed.1002898.s001.docx]

**S1 Table.**

**Satisfaction with the intervention**

| **Satisfaction with the single hospital consultation for both groups and for the additional 3 months complex occupational intervention after 6 months.** | | | | | | | |
| --- | --- | --- | --- | --- | --- | --- | --- |
|  | | **Intervention Arm** | | |  | **Comparison** | |
| **After 6 months** | | **No additional intervention (N=132)** | | **Occupational medicine**  **intervention (n=137)** |  | **P**  **Value** | |
| Satisfaction with usual care (1-5 scale) | | | |  |  | ^a^ 0.024 | |
|  | Very satisfied | 54 (41.2%) | | 71 (51.8%) |  |  |  |
|  | Overall satisfied | 37 (28.2%) | | 43 (31.4%) |  |  |  |
|  | Satisfied | 35 (26.7%) | | 15 (11.0%) |  |  |  |
|  | Not satisfied | 3 (2.3%) | | 4 (2.9%) |  |  |  |
|  | Very unsatisfied | 2 (1.5%) | | 4 (2.9% |  |  |  |
|  | Missing data | 1 | | 0 |  |  | |
| Satisfaction with the additional medicine intervention ^a^ (1-5 scale) | | |  |  |  |  |  |
|  | Very satisfied | NR | | 44 (34.4%) |  | NR | NR |
|  | Overall satisfied |  |  | 41 (32.0%) |  |  |  |
|  | Satisfied |  |  | 40 (31.3%) |  |  |  |
|  | Not satisfied |  |  | 3 (2.3%) |  |  |  |
|  | Very unsatisfied |  |  | 0 |  |  |  |
|  | Missing data |  | | 9 |  |  |  |
| Abbreviations: NR, not relevant. ^a^ Tested Chi-Squer test. NR= Not relevant. | | | | | | | |
