# Supplementary material for: Early occupational intervention for people with low back pain in physically demanding jobs: A randomized clinical trial
Source: PLoS Med. 2019 Aug 16;16(8):e1002898. doi: 10.1371/journal.pmed.1002898 (PMC6697316; doi:10.1371/journal.pmed.1002898)
Supplement: S2 Table — (DOCX) [file pmed.1002898.s002.docx]

**S2 Table. Additional analysis based on the available cases**

| **Available cases analyses. Changes in Primary and Secondary Outcomes for a Single Hospital Consultation with an Additional 3 months complex Occupational Oriented Intervention, Compared to a Single Hospital Consultation for Individuals in Physical Demanding Job and in Risk of Sick Leave (without drop-out data population).** | | | | | |
| --- | --- | --- | --- | --- | --- |
|  | **Intervention Arms** | |  | **Comparison** | |
| **Change from baseline to 6-month  follow-up** | **No additional intervention (N=132)** | **Occupational medicine**  **intervention (n=137)** |  | **Mean Changes Between Groups** | **P Value** |
| **Primary Outcome** |  |  |  |  |  |
| Cumulative self reported sick leave during 6 months from baseline, mean days (SD) | 15.22  (37.29) | 16.50  (37.69) |  | 0.12  (-7.90 to 8.15) | 0.976 |
| Cumulative self reported sick leave less than 7 days, n (%)* | 93  (70.5%) | 97  (70.8%) |  | OR=0.94  (0.53 to 1.66) | 0.822 |
| **Secondary Outcomes** |  |  |  |  |  |
| PDQ score for neuropathic pain | -2.43  (-3.95 to -0.91) | -1.26  (-2.73 to -0.22) |  | 1.09  (-0.09 to 2.27) | 0.071 |
| NRS pain intensity | -1.06  (-1.53 to -0.59) | -1.21  (-1.68 to -0.74) |  | 0.05  (-0.38 to 0.49) | 0.816 |
| RMDQ score for disability | -11.50  (- 16.97 to -6.02) | -11.11  (-16.73 to -5.48) |  | -1.71  (-5.41to 3.99) | 0.765 |
| FABQ score for physical activity | -2.24  (-3.47 to -1.02) | -2.80  (-4.09 to -1.50) |  | -0.53  (-1.66 to 0.61) | 0.363 |
| FABQ score for work | -2.74  (-4.76 to -0.72) | -2.82  (-4.89 to -0.76) |  | -0.10  (-1.89 to 1.69) | 0.911 |
| SF-36 physical component summary | 4.58  (2.60 to 6.55) | 4.17  (2.12 to 6.23) |  | -0.27  (-2.10 to 1.56) | 0.769 |
| SF-36 mental component summary | 2.00  (-1.61 to 4.60) | 1.55  (-1.15 to 4.25) |  | -0.28  (-2.73 to 2.17) | 0.821 |
| Self assessed ability to continue in work | 0.67  (0.22 to 1.11) | 0.66  (0.16 to 1.17) |  | 0.04  (-0.36 to 0.44) | 0.833 |
| Very satisfied or overall satisfied with the intervention | NR | 85 (66.4%) |  | NR | NR |
| Data are expressed as difference in means with 95% confidence intervals, unless otherwise indicated. The primary outcome is given as the number of days with sick leave in the 6 months after baseline. Secondary outcomes are given as mean change in the 6 months from baseline, and the comparison is given as the mean difference between groups in change from baseline. painDETECT questionnaire (PDQ) is a 0–30 scale (higher scores indicate a greater neuropathic components); numeric rating scale (NRS) is a 0–10 scale (higher scores indicate greater pain intensity); 24-item Roland–Morris Disability Questionnaire (RMDQ) is converted to a 0–100 score (higher scores indicate greater disability); Fear-Avoidance Beliefs Questionnaire (FABQ) is a 0–24 scale for physical activity (higher scores indicate greater fear-avoidance beliefs) and 0–42 scale for work (higher scores indicate greater fear-avoidance beliefs); Short Form Health Survey (SF-36) is a 0–100 scale for physical component summary (higher scores indicate higher physical function) and 0–100 scale for mental component summary (higher scores indicate higher mental health); ability to continue in work is assessed on a 0–10 scale (higher scores indicate better ability). *The comparison for this outcome is odds ratio (OR) instead of mean difference. | | | | | |
