# Supplementary material for: Early occupational intervention for people with low back pain in physically demanding jobs: A randomized clinical trial
Source: PLoS Med. 2019 Aug 16;16(8):e1002898. doi: 10.1371/journal.pmed.1002898 (PMC6697316; doi:10.1371/journal.pmed.1002898)
Supplement: S3 Table — (DOCX) [file pmed.1002898.s003.docx]

**S23 Table. Alternative imputation technique (BOCF)**

| **Imputation (Technique: Baseline carried forward)**  Comparison of Changes in Primary and Secondary Outcomes for a Single Hospital Consultation with an Additional 3 Months Complex Occupational Intervention, Compared to a Single Hospital Consultation for Individuals in Physical Demanding Job and in Risk of Sick Leave reanalyzed with Imputation. | | | | | |
| --- | --- | --- | --- | --- | --- |
|  | **Intervention Arm^a^** | |  | **Comparison** | |
| **Change from baseline to 6-months follow up.** | **No additional intervention (N=152^e^)** | **Additional Occupational**  **intervention (n=153^e^)** |  | **Mean Changes Between Groups** | **P Value** |
| **Primary Outcome^b^** |  |  |  |  |  |
| Cumulative self-reported sick-leave in days 6 months from baseline Mean days (SD) | NA | NA |  | NA | NA |
| Cumulative self-reported sick-leave less than 7 days, no. (%) | NA | NA |  | NA | NA |
| **Secondary Outcomes^c^** |  |  |  |  |  |
| PDQ score for neuropathic pain | -1.98  (-3.50 to -0.46) | -1.12  (-2.51 to 0.27) |  | 0.67  (-0.43 to 1.77) | 0.234 |
| NRS pain intensity | -0.91  (-1.38 to -0.45) | -1.08  (-1.53 to -0.64) |  | -0.07  (-0.48 to 0.33) | 0.722 |
| RMDQ score for disability | -9.61  (-14.94 to -4.28) | -9.95  (-15.34 to -4.56) |  | -1.30  (-5.67 to 3.06) | 0.558 |
| FABQ score for physical activity | -1.83  (-3.00 to -0.66) | -2.50  (-3.74 to -1.27) |  | -0.67  (-1.73 to 0.39) | 0.212 |
| FABQ score for work | -2.30  (-4.17 to -0.43) | -2.53  (-4.49 to -0.57) |  | -0.29  (-1.90 to 1.32) | 0.727 |
| SF-36 physical component summary | 3.91  (2.0 to 5.79) | 3.74  (1.79 to 5.68) |  | -0.02  (-1.69 to 1.66) | 0.984 |
| SF-36 mental component summary | 1.48  (-0.94 to 3.91) | 1.38  (-1.23 to 4.00) |  | -0.32  (-2.56 to 1.92) | 0.778 |
| Self-assessed ability to continue in work | 0.62  (0.19 to 1.05) | 0.71  (0.23 to 1.20) |  | 0.08  (-0.32 to 0.48) | 0.688 |
| Satisfaction with the intervention | NR | NR |  | NR | NR |
| Imputation based on 269 participants with complete data and 36 participants with baseline data. Data are expressed as difference in means with 95% confidence intervals, unless otherwise indicated. The primary outcome is given as the number of days with sick leave in the 6 months after baseline. Secondary outcomes are given as mean change in the 6 months from baseline, and the comparison is given as the mean difference between groups in change from baseline. painDETECT questionnaire (PDQ) is a 0–30 scale (higher scores indicate a greater neuropathic components); numeric rating scale (NRS) is a 0–10 scale (higher scores indicate greater pain intensity); 24-item Roland–Morris Disability Questionnaire (RMDQ) is converted to a 0–100 score (higher scores indicate greater disability); Fear-Avoidance Beliefs Questionnaire (FABQ) is a 0–24 scale for physical activity (higher scores indicate greater fear-avoidance beliefs) and 0–42 scale for work (higher scores indicate greater fear-avoidance beliefs); Short Form Health Survey (SF-36) is a 0–100 scale for physical component summary (higher scores indicate higher physical function) and 0–100 scale for mental component summary (higher scores indicate higher mental health); ability to continue in work is assessed on a 0–10 scale (higher scores indicate better ability). | | | | | |
