# Supplementary material for: Early occupational intervention for people with low back pain in physically demanding jobs: A randomized clinical trial
Source: PLoS Med. 2019 Aug 16;16(8):e1002898. doi: 10.1371/journal.pmed.1002898 (PMC6697316; doi:10.1371/journal.pmed.1002898)
Supplement: S4 Table — (DOCX) [file pmed.1002898.s004.docx]

**S4 Table. Baseline characteristics of the per-protocol and post-hoc analysis**

| **Baseline characteristics on the per-protocol analysis included only participants who received a workplace visit as part the additional 3 months occupational intervention.** | | | | | | |
| --- | --- | --- | --- | --- | --- | --- |
|  | | | | **No additional intervention (n=152)** | **Additional Occupational Intervention (n=40)** | **All Participants**  **(n=192)** |
| **Sociodemographic Characteristics** | | | |  |  |  |
|  | Female sex | | | 50 (32.9%) | 16 (40.0%) | 66 (34.4%) |
|  | Age, y, | | | 45.7 (±10.5) | 47.8 (±10.0) | 46.2 (±10.4) |
|  | Current smoker | | | 63 (41.5%) | 13 (32.5%) | 76 (39.6%) |
|  | BMI, kg/m^2^ | | | 16.3 (±13.5) | 15.9 (±15.6) | 16.3 (±13.8) |
|  | >9 years education | | | 112 (73.7%) | 27 (67.5%) | 139 (72.4%) |
| **Employment Characteristics** | | | |  |  |  |
|  | Job category DISCO-88 code >5 | | | 83 (54.6%) | 27 (67.5%) | 110 (57.3%) |
|  | Self-assessed workability (0-10) | | | 6.23 (1.87) | 6.08 (1.98) | 6.20 (1.89) |
|  | Self reported current physical workload | | |  |  |  |
|  |  | | Very demanding | 75 (49.3%) | 15 (37.5%) | 90 (46.9%) |
|  |  | | Demanding | 73 (48.0%) | 25 (62.5%) | 98 (51.0%) |
|  |  | | Medium demanding | 4 (2.6%) | 0 (0%) | 7 (2.1%) |
|  | Sick leave due to LBP last year >7 days | | | 68 (44.7%) | 18 (45.0%) | 86 (44.7%) |
|  | Sick leave due to LBP last year >1 months | | | 17 (11.2%) | 5 (12.5%) | 22 (11.5%) |
| **Low back pain Characteristics** | | | |  |  |  |
|  | Duration of LBP ≥ 3 months | | | 110 (72.4%) | 33 (82.5%) | 143 (74.5%) |
|  | NRS for pain | | | 5.5 (1.98) | 5.7 (1.9) | 5.6 (2.0) |
|  | LBP without sciatica / radiculopathy | | | 84 (55.3%) | 18 (45.0%) | 102 (53.1%) |
|  | Neurologic deficit | | | 23 (15.1%) | 9 (22.5%) | 32 (16.7%) |
|  | PDQ | | | 11.9 (6.5) | 11.7 (5.7) | 11.9 (6.4) |
|  | RMDQ | | | 51.5 (22.7) | 52.6 (20.8) | 51.7 (22.3) |
|  | FABQ - Physical activity | | | 14.6 (5.0) | 14.9 (4.6) | 14.7 (4.9) |
|  | FABQ - Work | | | 25.2 (7.3) | 24.7 (7.7) | 25.1 (7.3) |
| **Current medications for back pain** **(weekly)** | | | | |  |  |
|  | Nonsteroidal anti-inflammatory | | | 109 (71.7%) | 32 (80.0%) | 141 (73.4%) |
|  | Opioid | | | 32 (21.1%) | 12 (30.0%) | 44 (22.9%) |
|  | Muscle relaxers/Anticonvulsant | | | 5 (3.3%) | 0 (0%) | 5 (2.6%) |
|  | Steroid anti-inflammatory | | | 3 (2.0%) | 2 (5.0%) | 5 (2.6%) |
|  | Other (e.g. acetaminophen) | | | 134 (88.2%) | 38 (95.0%) | 172 (89.5%) |
| **Health Related Quality of Life (HR – QoL)** | | | |  |  |  |
|  | SF-36 – PCS for physical functional | | | 37.7 (7.6) | 35.9 (7.0) | 37.3 (7.5) |
|  | SF-36 - MCS for mental health | | | 48.4 (10.9) | 46.4 (11.2) | 48.0 (11.0) |
| **Magnetic resonance imaging*** | | | |  |  |  |
|  | | Herniation | | 77 (52.0%) | 18 (46.2%) | 95 (50.8%) |
|  | | Spinal stenosis | | 25 (16.9%) | 6 (15.4%) | 31 (16.6%) |
|  | | Inflammatory spinal disease | | 7 (4.8%) | 0 (0%) | 7 (3.7%) |
|  | | Spondylolistesis | | 17 (11.5%) | 6 (15.4%) | 23 (12.3%) |
|  | | Non-specific spondylosis | | 83 (56.1%) | 27 (69.2%) | 110 (58.8%) |
|  | | Morbus Scheuermann stigmata | | 7 (4.8%) | 1 (2.6%) | 8 (4.3%) |
| Data are given as *n* (%) or mean (SD). For the Danish version of the International Standard Classification of Occupations (DISCO-88), categories range from 1 to 9, with job categories over 5 indicating manual labor and/or physical work (blue-collar workers). Ability to stay in job was assessed on a 0 to 10 scale, with higher scores indicating better ability to stay in job. For the numeric rating scale (NRS) for pain, scores range from 0 to 10, with higher scores indicating more pain. For the painDETECT questionnaire (PDQ), scores range from 0 to 30, with higher scores indicating more neuropathic pain. For the Roland–Morris Disability Questionnaire (RMDQ), scores range from 0 to 100 after converting from a 24-item scale to a 23-item scale, with higher scores indicating greater disability. For the Fear-Avoidance Beliefs Questionnaire (FABQ) subscale for physical activity, the scores range from 0 to 24, with higher scores indicating greater fear-avoidance beliefs towards physical activities, and for the subscale for work, the scores range from 0 to 42, with higher scores indicating greater fear-avoidance beliefs towards work. For the Short Form Health Survey (SF-36) physical component summary (PCS), the scores range from 0 to 100, with higher scores indicating better physical function, and for the mental composite summary (MCS), the scores range from 0 to 100, with higher scores indicating better mental health. LBP, low back pain. | | | | | | |

| **Baseline characteristics on the post-hoc analysis including participants who at baseline assessment reported that their job was very demanding.** | | | | | | |
| --- | --- | --- | --- | --- | --- | --- |
|  | | | | **No additional intervention (n=75)** | **Additional**  **Occupational Oriented Intervention (n=69)** | **All Participants**  **(n=144)** |
| **Sociodemographic Characteristics** | | | |  |  |  |
|  | Female sex | | | 24 (32%) | 24 (34.8%) | 48 (33.3%) |
|  | Age, years | | | 43.3 (11.2) | 43.3 (9.4) | 43.3 (10.4) |
|  | Current smoker | | | 46 (61.3%) | 46 (66.7%) | 92 (63.9%) |
|  | BMI kg/m^2^ | | | 15.8 (13.0) | 16.7 (14.6) | 16.3 (13.7) |
|  | >9 y education | | | 57 (76.0%) | 43 (62.3%) | 100 (69.4%) |
| **Employment Characteristics** | | | |  |  |  |
|  | Job category DISCO-88 code >5 | | | 45 (60.0%) | 43 (62.3%) | 88 (61.1%) |
|  | Self-assessed workability^c^ (0-10) | | | 6.17 (2.02) | 5.78 (2.48) | 5.99 (2.25) |
|  | Self reported current physical workload | | |  |  |  |
|  |  | | Very demanding | 75 (100%) | 69 (100%) | 144 (100%) |
|  |  | | Demanding | NR | NR | NR |
|  |  | | Medium demanding | NR | NR | NR |
|  | Sick leave due to LBP last year >7 days | | | 37 (49.3%) | 38 (55.1%) | 75 (52.1%) |
|  | Sick leave due to LBP last year >1 months | | | 8 (10.7%) | 11 (15.9%) | 19 (13.2%) |
| **Low back pain Characteristics** | | | |  |  |  |
|  | Duration of LBP ≥ 3 months | | | 62 (82.7%) | 66 (95.7%) | 128 (88.9%) |
|  | NRS for pain | | | 5.8 (2.0) | 6.2 (1.8) | 6.0 (1.9) |
|  | LBP without sciatica / radiculopathy | | | 44 (58.7%) | 41 (59.4%) | 85 (59.0%) |
|  | Neurologic deficit | | | 11 (14.7%) | 12 (17.4%) | 23 (16.0%) |
|  | PDQ | | | 12.3 (7.2) | 12.0 (6.7) | 12.0 (7.0) |
|  | RMDQ | | | 52.6 (24.0) | 51.4 (20.6) | 52.0 (22.4) |
|  | FABQ - Physical activity | | | 15.3 (4.8) | 16.2 (4.5) | 15.7 (4.6) |
|  | FABQ - Work | | | 27.4 (6.9) | 28.2 (7.0) | 27.8 (6.9) |
| **Current medications for back pain** **(weekly)** | | | | |  |  |
|  | Nonsteroidal anti-inflammatory | | | 51 (68.0%) | 50 (72.5%) | 101 (70.1%) |
|  | Opioid | | | 14 (18.7%) | 16 (23.2%) | 30 (20.8%) |
|  | Muscle relaxers/Anticonvulsant | | | 2 (2.7%) | 0 (0%) | 2 (1.4%) |
|  | Steroid anti-inflammatory | | | 2 (2.7%) | 2 (2.9%) | 4 (2.8%) |
|  | Other (e.g. acetaminophen) | | | 66 (88.0%) | 66 (95.7%) | 132 (91.6%) |
| **Health Related Quality of Life (HR – QoL)** | | | |  |  |  |
|  | SF-36 – PCS for physical functional | | | 37.1 (7.8) | 36.4 (7.2) | 36.8 (7.5) |
|  | SF-36 - MCS for mental health | | | 48.6 (10.9) | 46.4 (10.6) | 47.5 (10.8) |
| **Magnetic resonance imaging*** | | | |  |  |  |
|  | | Herniation | | 36 (50.0%) | 31 (46.7%) | 67 (48.6%) |
|  | | Spinal stenosis | | 9 (12.5%) | 7 (10.6%) | 16 (11.6%) |
|  | | Inflammatory spinal disease | | 6 (8.3%) | 2 (3.0%) | 7 (5.8%) |
|  | | Spondylolistesis | | 7 (9.7%) | 13 (19.7%) | 20 (14.5%) |
|  | | Non-specific spondylosis | | 36 (50.0%) | 37 (56.1%) | 73 (52.9%) |
|  | | Morbus Scheuermann stigmata | | 3 (4.2%) | 7 (10.6%) | 10 (7.2%) |
| Data are given as *n* (%) or mean (SD). For the Danish version of the International Standard Classification of Occupations (DISCO-88), categories range from 1 to 9, with job categories over 5 indicating manual labor and/or physical work (blue-collar workers). Ability to stay in job was assessed on a 0 to 10 scale, with higher scores indicating better ability to stay in job. For the numeric rating scale (NRS) for pain, scores range from 0 to 10, with higher scores indicating more pain. For the painDETECT questionnaire (PDQ), scores range from 0 to 30, with higher scores indicating more neuropathic pain. For the Roland–Morris Disability Questionnaire (RMDQ), scores range from 0 to 100 after converting from a 24-item scale to a 23-item scale, with higher scores indicating greater disability. For the Fear-Avoidance Beliefs Questionnaire (FABQ) subscale for physical activity, the scores range from 0 to 24, with higher scores indicating greater fear-avoidance beliefs towards physical activities, and for the subscale for work, the scores range from 0 to 42, with higher scores indicating greater fear-avoidance beliefs towards work. For the Short Form Health Survey (SF-36) physical component summary (PCS), the scores range from 0 to 100, with higher scores indicating better physical function, and for the mental composite summary (MCS), the scores range from 0 to 100, with higher scores indicating better mental health. LBP, low back pain. | | | | | | |
