# Supplementary material for: Early occupational intervention for people with low back pain in physically demanding jobs: A randomized clinical trial
Source: PLoS Med. 2019 Aug 16;16(8):e1002898. doi: 10.1371/journal.pmed.1002898 (PMC6697316; doi:10.1371/journal.pmed.1002898)
Supplement: S1 Text — (PDF) [file pmed.1002898.s005.pdf]

STATISTICAL ANALYSIS PLAN (SAP):

Retention in physically demanding jobs of individuals with low back pain: SAP for a single center, randomized clinical superiority trial

Version: October 9st, 2017

COLLABORATORS:

Bjarke B. Hansen, Lilli Kirkeskov, Robin Christensen, Luise M. Begtrup, Mikael Boesen, Gilles Ludger Fournier, Ditte Lundsgaard Anderasen, Lars Erik Kristensen, Henning Bliddal, Esben M. Flachs, and Ann I. Kryger.

Original Published Protocol:

Hansen BB, Kirkeskov L, Christensen R, Begtrup LM, Pedersen EB, Teilya JF, Boesen M, Fournier GL, Bliddal H, Kryger AI. Retention in physically demanding jobs of individuals with low back pain: study protocol for a randomized controlled trial. Trials. 2015 Apr 16;16:166. doi: 10.1186/s13063-015-0684-3.

Statistical Analyst:

Esben Meulengracht Flachs, MSc, PhD; Biostatistician

Department of Occupational and Environmental Medicine, Copenhagen University Hospital, Bispebjerg and Frederiksberg, Bispebjerg Bakke 23, DK-2400 Copenhagen, NV, Denmark.

Statistical Advisor:

Dr. Robin Christensen, MSc, PhD; Biostatistician

Professor of Clinical Epidemiology, adj.

Head of Musculoskeletal Statistics Unit, The Parker Institute, Bispebjerg and Frederiksberg Hospital, DK-2000 Copenhagen F, Denmark.

ORIGINAL PROTOCOL SUMMARY

**Protocol title**	Retention in physically demanding jobs of individuals with low back pain: study protocol for a randomised controlled trial
**Protocol publication 2015**	**Trials. 2015** Apr 16:166. doi: 10.1186/s13063-015-0684-3.
**Introduction**	Low back pain is a frequent cause of disability and sick leave among working adults. Individuals with low back pain frequently consult general practice or other health care providers, which often results in a unilateral intervention focused on their symptoms. Employment is associated with physical and mental well-being, so, patients may benefit from an early additional occupational medicine intervention. For individuals with physically demanding jobs it can be especially challenging to retain their jobs. Therefore, it is necessary to develop and study interventions with the aim to retain people in work, despite physical demanding jobs.
**Aim**	The aim of the 'GoBack trial' is to develop and evaluate the efficacy and feasibility of an occupational medicine intervention for individuals with low back pain in physically demanding jobs.
**Trial registration**	ClinicalTrials.gov (identifier: NCT02015572) on 29 November 2013.
**Methods/design**	We will conduct a randomized controlled trial enrolling people with difficulty in maintaining physically demanding jobs due to low back pain for a current period of 2 to 4 weeks. Participants will be randomized (1:1; stratified according to age and gender) before being allocated either to additional occupational medicine intervention or control.
**Inclusion criteria**	The study population will consist of individuals: 1) 18-65 years of age; 2) current episode of 2-4 weeks of low back pain; 3) self-reported physically demanding work; 4) express concerns about the ability to maintain their current job.
**Exclusion criteria**	Candidates will be excluded due to: 1) pregnancy; 2) severe somatic or psychiatric diseases; 3) cancer or metastatic disease.
**Primary outcome**	Accumulated duration of self-assessed sick leave (in days) due to low back pain during 6 months from baseline.
**Secondary outcomes**	Changes in pain level; (*ii*) Changes in Fear Avoidance Beliefs scores, (*iii*) Change in Disability; and (*iv*) Satisfaction with the intervention [6 months from baseline].
**Intervention**	All participants will receive conventional medical care. The study's additional occupational medicine intervention will last 3 months and include: 1) consultations with occupational medicine physician; 2) a workplace visit, if required; 3) a consultation with a physiotherapist; 4) a weekly telephone interview with focus on adherence to the intervention plan; 5) midway interview with focus on return to/retention at work; 6) a three months a session concluding further guidance.
**Power calculation**	A sample size of 127 participants per group is required to obtain a power of at least 80% to detect a mean difference in sick leave of 6 days. Expecting some drop-outs during the trial period (less than 20%), it was decided for pragmatic reasons to enroll 300 participants in total.

**Ethical aspects**	All participants are insured according to the national health insurance and we will follow the guidance in the approval by the Local Research Ethics Committee, Region H, Denmark (H-3-2013-161) 20 November 2013
**Risks and disadvantages**	All participants will receive usual standard care, and no treatment will be withheld to participants in this trial. Both allocation groups receive active treatments with previously demonstrated efficacy and no iatrogenic effects.
**Rational**	The findings from this randomised trial will provide high-quality evidence for the efficacy and feasibility of an occupational medicine intervention model for individuals with low back pain in physically demanding jobs.

PURPOSE OF THE STATISTICAL ANALYSIS PLAN (SAP)

The SAP is intended to bring the team together on the same page. For biostatisticians and methodologists, all inferential analyses and statistical methods are delineated. For programmers, the SAP hopefully provides explicit guidance on the various SAS/R codes. Also, the SAP outlines the pre-specified format for the presentation of the figures, tables, important appendices, and listings. Thus, the SAP adds another layer of specificity to the project. The SAP was finalized before breaking the blind (i.e., having access to data). Formal records will be kept of when the SAP was finalized as well as when the blind was subsequently broken.

INTRODUCTION

Low back pain (LBP) is a recognized public health problem with high life time prevalence. Medical treatment may reduce the physical and mental discomfort, while it has not been able to improve the possibilities for retaining or return patients with LBP to work.

This is an occupational intervention study for patients with LBP and physically demanding work, who are at risk of drop out of labor; a randomized clinical trial designed to test the effectiveness of an early intervention for retaining subjects with LBP attached to the labor marked. A work place modification intervention combined with moderate physical activity is given in the intervention group additional to LBP treatments according to best practice recommendations for general practice.

The study population consists of patients in self-reported physically demanding jobs, which are sick listed or at risk of sick leave due to LBP. Outcome will continually be collected during the intervention as well as 6 months follow up.

The primary aim is to evaluate if an occupational intervention with focus on early workplace orientated counselling and work place intervention can retain subjects with physically demanding work and LBP in gainful employment.

DATA SOURCE

Eligible participants were allocated according to the randomization after baseline measurements to one of two parallel groups: 1) control, 2) additional occupational medicine intervention. Data were collected at each stage of the trial, at initial telephone screening, allocation, occupational sessions and at the follow-up visit.

Eligible participants were: 1) 18-65 years of age; 2) current episode of 2-4 weeks of LBP; 3) self-reported physically demanding work; 4) express concerns about the ability to maintain their current job. The exclusion criteria were: 1) pregnancy; 2) server somatic or psychiatric diseases; 3) cancer or metastatic disease. The study was approved by the Local Research Ethics Committee, Region H, Denmark (H-3-2013-161) 20 November 2013; NCT02015572.

ANALYSIS OBJECTIVES

The study is designed to investigate if an additional occupational medicine intervention can retain individuals with physically demanding jobs and LBP in gainful employment over a 6-month period.

Between-group comparison of the primary end point (the accumulated duration of self-assessed sick leave [in days] will be made with the use of analysis of covariance; a confidence interval excluding differences greater than 6 days between groups will be interpreted as indicating the absence of a clinically meaningful difference (Bland JM. The tyranny of power: is there a better way to calculate sample size? BMJ. 2009 Oct 6;339:b3985).

ANALYSIS SETS/POPULATIONS/SUBGROUPS

Primary 'Full Analysis Set': Statistical analyses will be performed on the ITT population, including all randomized participants. Missing data will be replaced using multiple imputation (5 iterations), including age, sex, baseline level (sick leave due to LBP the last year [days]), and group allocations (blinded) as predictors.

'As Observed' Analysis Set: Including all randomized participants; missing data will not be replaced.

'Per Protocol' Analysis Set: Including all randomized participants, who had a formal working place visit AND who completed the 6 months sick leave diary (for control-participants: only the latter

apply). We will explore whether there is a more pronounced (better) effect among the *Per protocol* individuals compared to those in the Full Analysis Set (ITT population).

ENDPOINTS AND COVARIATES

Outcome measures and variables informative of context are presented below (see Table 1 and Table 2).

HANDLING OF MISSING VALUES AND OTHER DATA CONVENTIONS

If all participants randomized into the GO-BACK clinical trial satisfied all entry criteria, followed all trial procedures perfectly with no losses to follow-up, and provided complete data records, then the set of subjects to be included in the analysis would be self-evident.

For each group, the numbers of participants who were randomly assigned, received intended treatment, and were analyzed for the primary outcome will be presented in a participant flow diagram (see example in Figure 1).

It is considered advantageous to demonstrate a lack of sensitivity of the principal trial results to alternative choices of the set of participants analyzed. Thus, we will conduct both an analysis of the full analysis set (the ITT population) and a *per protocol* analysis, so that any differences between them can be the subject to explicit discussion and interpretation.

When the full analysis set and the per protocol set lead to essentially the same conclusions, confidence in the trial results will be increased, bearing in mind, however, that the need to exclude a substantial proportion of subjects from the per protocol analysis throws some doubt on the overall validity of the trial.

STATISTICAL PROCEDURES

For continuous outcomes (incl. the primary endpoint), an Analysis of Covariance (ANCOVA) will be applied: the model include treatment group (I vs C), age group (<40 or ≥ 40 years), sex (Male vs Female), and the baseline value of the relevant variable as a covariate. Categorical changes for dichotomous end points will be analyzed with the use of logistic regression with the same covariates as the respective analysis of covariance.

MEASURES TO ADJUST FOR MULTIPLICITY, CONFOUNDERS, HETEROGENEITY

Outcome measures are, often systematically related to other influences apart from treatment. For example, there may be relationships to covariates such as age and sex. In the primary model we will adjust for the influence of covariates that were used for stratification (sex and age) as set out in the protocol.

As these two factors were used to stratify the design, it is appropriate to account for those factors in the analysis. According to the ICH E9 guidance, special attention should be paid to the role of baseline measurements of the primary variable. We will NOT adjust the main analyses for covariates measured after group allocation because they may be affected (mediated) by the treatment.

SENSITIVITY ANALYSES

Bias might arise during the conduct and analysis of a clinical trial. For example, protocol violations and exclusion of subjects from analysis based upon knowledge of the participants outcomes are possible source of bias that may affect the accurate assessment of the treatment effect. Because bias can occur in subtle or unknown ways and its effect is not measurable directly, it is important to evaluate the robustness of the results and primary conclusions of the trial.

Robustness is a concept that refers to the sensitivity of the overall conclusions to various limitations of the data, assumptions, and analytic approaches to data analysis. Robustness implies that the treatment effect and primary conclusions of the trial are not substantially affected when analyses are carried out based on alternative assumptions or analytic approaches. The interpretation of statistical measures of uncertainty of the treatment effect and treatment comparisons should involve consideration of the potential contribution of bias to the 95% confidence interval, or inference.

Sensitivity analyses will be performed in the GO-BACK trial to assess the robustness of the primary analyses, including worst- and best-case scenarios (missing data manually replaced with the each of the options).

RATIONALE FOR ANY DEVIATION FROM PRE-SPECIFIED PROTOCOL

None.

PROPOSED MANUSCRIPT OUTLINE (FIGURES, TABLES, APPENDICES)

Figure 1: Flow diagram (study profile)

Table 1: Baseline characteristics

Figure 2: Illustration of the primary analysis

Table 2: Change from baseline for all clinical outcomes (Multiple imputation)

Table 3: Harms and adverse events (or in text)

Appendix 1: The Final Statistical Analysis Plan (SAP)

Appendix 2: As Table 2; based on the as observed population (no imputations)

Appendix 3: As Table 2; including only the Per Protocol Population.

Figure 1| Flow diagram will be conducted according CONSORT guideline and include the progress through the phases of the parallel randomized trial of two groups (that is, enrolment, intervention allocation, follow-up, and data analysis)

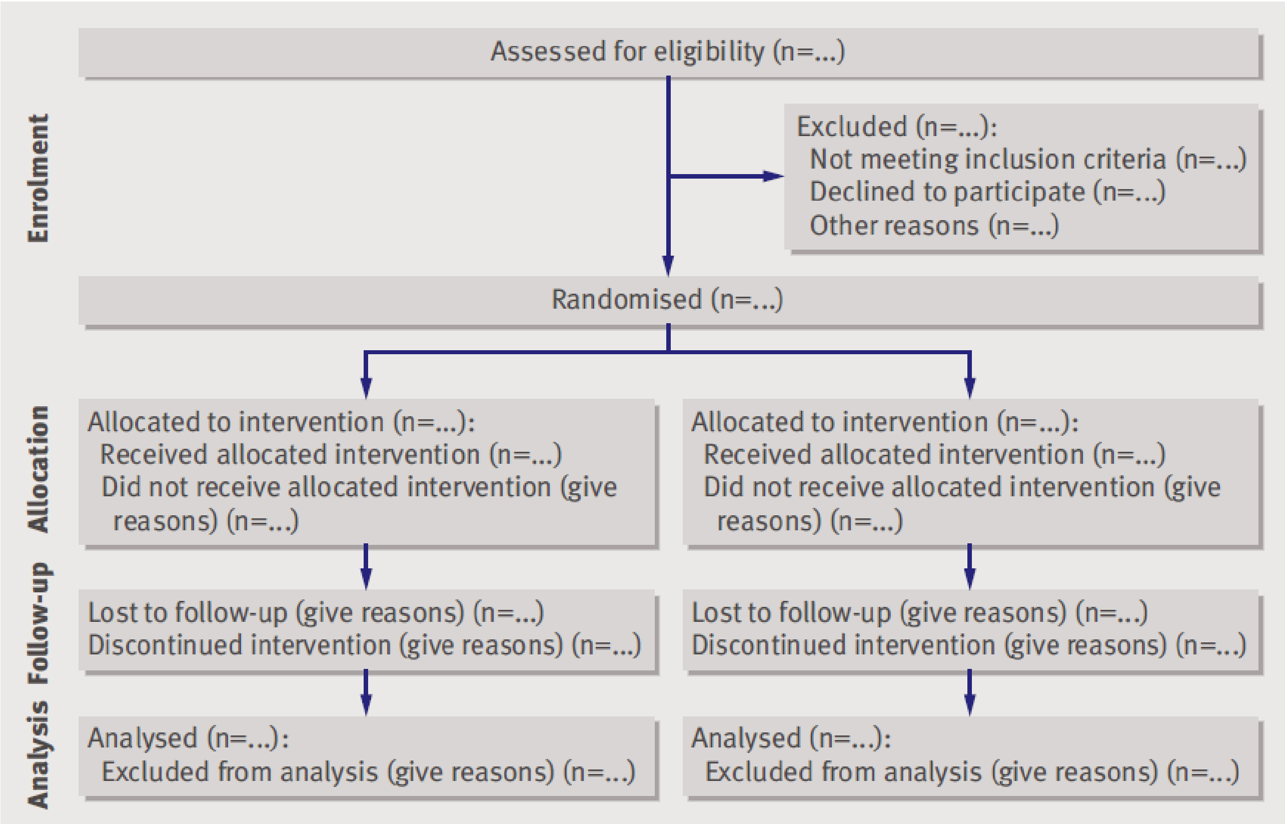

Table 1. Baseline demographic and clinical characteristics

	No (%)[a]		
	Usual care (n = XXX)	Occupational medicine Intervention (n = XXX)	All Participants (n = XXX)
**Sociodemographic Characteristics**			
Female, sex			
Age, y, Mean (SD)			
Current smoker			
Weight, kg, Mean (SD)			
Height, m, Mean (SD)			
>Primary school (>9 y education)			
**Employment Characteristics**			
Job category, disco code			
Workability			
Physically work load graded (1-4) Median (IQR)			
Sick leave the last year, d, No.			
**Low back pain Characteristics**			
Duration of LBP ≥ 3 months			
Pain numeric rating scale, mean (SD)			
LBP without sciatica / radiculopathy			
Neurologic deficit			
PDQ, mean (SD)			
RMDQ, mean (SD)			
FABQ - Physical activity, mean (SD)			
FABQ - Work, mean (SD)			
**Current medications for back pain**			
Nonsteroidal anti-inflammatory			
Opioids			
Muscle relaxers			
Steroid anti-inflammatory			
Other			
**Patient health**			
SF-36 - physical functional, mean (SD)			
SF-36 - Mental health, mean (SD)			
**Magnetic resonance imaging**			
Herniation**			
Spinal stenosis			
Spondylolistesis			
Inflammatory spinal disease			
Non-specific spondylosis			

Abbreviations: BMI, body mass index (calculated as weight in kilograms divided by height in meters squared); PDQ, Pain-Detect Questionnaire; FABQ, Fear-Avoidance Beliefs Questionnaire; RMDQ, the 24-item Roland Morris Disability Questionnaire; SF-36, Short-Form questionnaire; **Herniation (i.e. Protrusion, extrusions or bulging) [a] Unless otherwise indicated.

Figure 2. Effect on accumulated duration of self-assessed sick leave due to LBP (days; primary outcome measure and analysis) Example given:

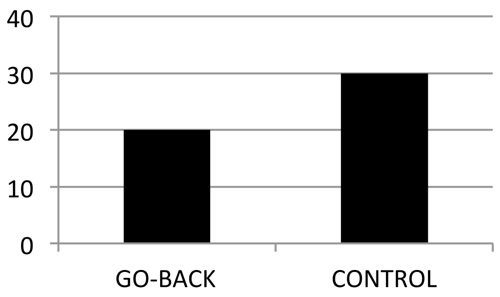

Values are Least Squares Means (95% Confidence Intervals); Based on Multiple imputation Technique.

Table 2. Comparison of Changes in Primary and Secondary Outcomes for occupational medicine intervention Usual Care for individuals in risk of sick leave and in physical demanding job.

	**Usual Care**	**Occupational medicine intervention**	**Comparison**	
Outcome	Mean Change From Baseline	Mean Change From Baseline*	Mean Difference Between Groups	P Value
**Primary Outcome**				
Cumulative self-reported sick-leave				
**Secondary Outcomes**				
PDQ score (0-30 scale; higher scores indicate a greater neuropathic components)				
Numeric pain rating (0-10 scale; higher scores indicate greater pain intensity)				
RMDQ score for disability (converted to a 0-100 score; higher scores indicate greater disability)				
FABQ score for physical activity (0-24 scale; higher scores indicate greater fear-avoidance beliefs)				

FABQ score for work (0-42 scale; higher scores indicate greater fear-avoidance beliefs)			
Satisfaction with the intervention Numeric rating scale (0-10 scale; higher scores indicate greater Satisfaction			
SF-36 for physical component score (0-100 scale; higher scores indicate higher physical function)§			
SF-36 score for mental health (0-100 scale; higher scores indicate higher mental health§			
Work Ability Index questionnaire§			

Values are Least-Squares Means with 95% Confidence Intervals. Abbreviations: PDQ, Pain-Detect Questionnaire; FABQ, Fear-Avoidance Beliefs Questionnaire; RMDQ, the 24-item Roland Morris Disability Questionnaire; SF-36, Short-Form questionnaire.

§ Secondary Exploratory Outcomes (not registered in Clinicaltrials.gov)

Harms and adverse events will be reported in text or in the following table

Table 3. Observed harms and adverse events in the ITT population

Outcome	Intervention	Control	Difference
Patients with any Adverse Event, no.(%)			
Patients with any Serious Adverse Event, no. (%)			
Deaths, no. (%)			
Total number of Adverse Events			
Total number of Patients-Years			
Total number of Serious Adverse Events			
