# Supplementary material for: Early occupational intervention for people with low back pain in physically demanding jobs: A randomized clinical trial
Source: PLoS Med. 2019 Aug 16;16(8):e1002898. doi: 10.1371/journal.pmed.1002898 (PMC6697316; doi:10.1371/journal.pmed.1002898)
Supplement: S2 Text — (DOC) [file pmed.1002898.s006.doc]

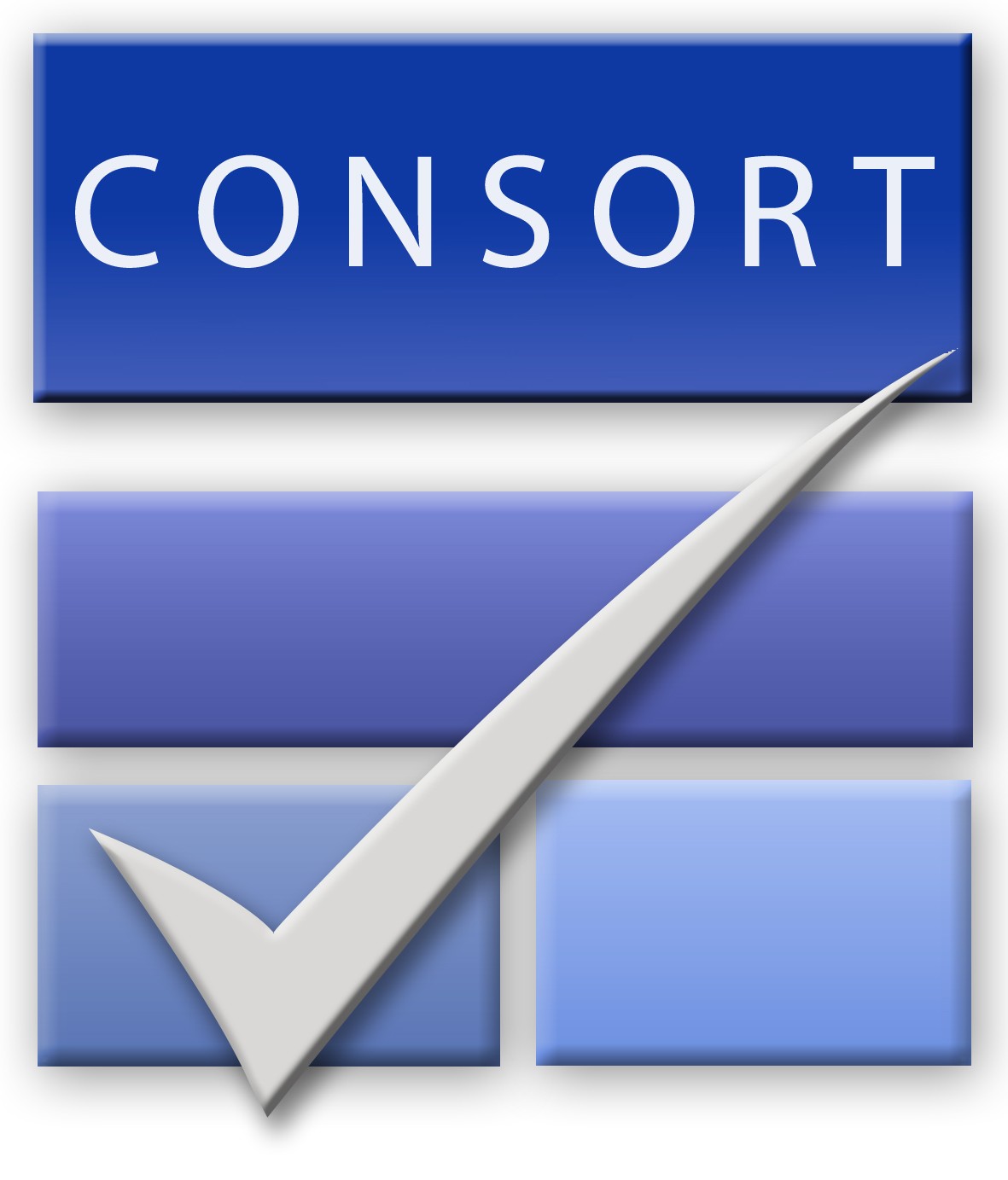
CONSORT 2010 checklist of information to include when reporting a randomised trial*

| Section/Topic | Item No | Checklist item | Reported on page No |
| --- | --- | --- | --- |
| Title and abstract | | | |
|  | 1a | Identification as a randomised trial in the title | See Title |
|  | 1b | Structured summary of trial design, methods, results, and conclusions (for specific guidance see CONSORT for abstracts) | See Author Summary and abstract |
| Introduction | | | |
| Background and objectives | 2a | Scientific background and explanation of rationale | See Introduction |
|  | 2b | Specific objectives or hypotheses | See Introduction |
| Methods | | | |
| Trial design | 3a | Description of trial design (such as parallel, factorial) including allocation ratio | See Methods paragraphs 1 in Design Setting and participants and Randomization |
|  | 3b | Important changes to methods after trial commencement (such as eligibility criteria), with reasons | N/A |
| Participants | 4a | Eligibility criteria for participants | See Methods paragraphs 2 in Design Setting and participants |
|  | 4b | Settings and locations where the data were collected | See Methods paragraphs 1 in Design Setting and participants |
| Interventions | 5 | The interventions for each group with sufficient details to allow replication, including how and when they were actually administered | See Intervention |
| Outcomes | 6a | Completely defined pre-specified primary and secondary outcome measures, including how and when they were assessed | See Outcome assessments |
|  | 6b | Any changes to trial outcomes after the trial commenced, with reasons | N/A |
| Sample size | 7a | How sample size was determined | See Sample size and statistical analysis, paragraph 1 |
|  | 7b | When applicable, explanation of any interim analyses and stopping guidelines | N/A |
| Randomisation: |  |  |  |
| Sequence generation | 8a | Method used to generate the random allocation sequence | See Sample size and statistical analysis, paragraph 1 |
|  | 8b | Type of randomisation; details of any restriction (such as blocking and block size) | See Sample size and statistical analysis, paragraph 1 |
| Allocation concealment mechanism | 9 | Mechanism used to implement the random allocation sequence (such as sequentially numbered containers), describing any steps taken to conceal the sequence until interventions were assigned | See Sample size and statistical analysis, paragraph 1 |
| Implementation | 10 | Who generated the random allocation sequence, who enrolled participants, and who assigned participants to interventions | See Sample size and statistical analysis, paragraph 1, |
| Blinding | 11a | If done, who was blinded after assignment to interventions (for example, participants, care providers, those assessing outcomes) and how | N/A |
|  | 11b | If relevant, description of the similarity of interventions | See Intervention |
| Statistical methods | 12a | Statistical methods used to compare groups for primary and secondary outcomes | See Sample size and statistical analysis, paragraph 2 |
|  | 12b | Methods for additional analyses, such as subgroup analyses and adjusted analyses | See Sample size and statistical analysis, paragraph 2 |
| Results | | | |
| Participant flow (a diagram is strongly recommended) | 13a | For each group, the numbers of participants who were randomly assigned, received intended treatment, and were analysed for the primary outcome | See Results, Baseline Characteristics |
|  | 13b | For each group, losses and exclusions after randomisation, together with reasons | See Results, Baseline Characteristics and Figure 1 |
| Recruitment | 14a | Dates defining the periods of recruitment and follow-up | See Results, Baseline Characteristics |
|  | 14b | Why the trial ended or was stopped | N/A |
| Baseline data | 15 | A table showing baseline demographic and clinical characteristics for each group | See Table 1 |
| Numbers analysed | 16 | For each group, number of participants (denominator) included in each analysis and whether the analysis was by original assigned groups | See Results, Adherence to the intervention and Table 1 |
| Outcomes and estimation | 17a | For each primary and secondary outcome, results for each group, and the estimated effect size and its precision (such as 95% confidence interval) | See Results, Primary and secondary outcomes  and table 2 |
|  | 17b | For binary outcomes, presentation of both absolute and relative effect sizes is recommended | N/A |
| Ancillary analyses | 18 | Results of any other analyses performed, including subgroup analyses and adjusted analyses, distinguishing pre-specified from exploratory | See Results, Sensitivity analyses and table 3 and 4 |
| Harms | 19 | All important harms or unintended effects in each group (for specific guidance see CONSORT for harms) | See Results, Adherence to the intervention, paragraph 2 |
| Discussion | | | |
| Limitations | 20 | Trial limitations, addressing sources of potential bias, imprecision, and, if relevant, multiplicity of analyses | See discussion paragraph 6 |
| Generalisability | 21 | Generalisability (external validity, applicability) of the trial findings | See discussion paragraph 2,3,4,5, |
| Interpretation | 22 | Interpretation consistent with results, balancing benefits and harms, and considering other relevant evidence | See discussion paragraph 2,3,4,5, |
| Other information | | |  |
| Registration | 23 | Registration number and name of trial registry | See Sample size and statistical analysis, paragraph 2 |
| Protocol | 24 | Where the full trial protocol can be accessed, if available | See Methods paragraphs 1 in Design Setting and participants |
| Funding | 25 | Sources of funding and other support (such as supply of drugs), role of funders | See Acknowledgments |

*We strongly recommend reading this statement in conjunction with the CONSORT 2010 Explanation and Elaboration for important clarifications on all the items. If relevant, we also recommend reading CONSORT extensions for cluster randomised trials, non-inferiority and equivalence trials, non-pharmacological treatments, herbal interventions, and pragmatic trials. Additional extensions are forthcoming: for those and for up to date references relevant to this checklist, see [www.consort-statement.org](http://www.consort-statement.org/).
