# Supplementary material for: Early occupational intervention for people with low back pain in physically demanding jobs: A randomized clinical trial
Source: PLoS Med. 2019 Aug 16;16(8):e1002898. doi: 10.1371/journal.pmed.1002898 (PMC6697316; doi:10.1371/journal.pmed.1002898)
Supplement: S3 Text — (PDF) [file pmed.1002898.s007.pdf]

ClinicalTrials.gov
Protocol Registration System

Protocol Registration Receipt
12/13/2013

Retention in Physically Demanding Jobs With Low Back Pain: A Randomised Controlled Trial (GoBack)

This study is not yet open for participant recruitment.
Verified by Bjarke Brandt Hansen, Frederiksberg University Hospital, December 2013

Sponsor:	Frederiksberg University Hospital
Collaborators:	Bispebjerg Hospital
Information provided by (Responsible Party):	Bjarke Brandt Hansen, Frederiksberg University Hospital
ClinicalTrials.gov Identifier:	

Purpose

Low back pain (LBP) is a recognized public health problem with high life time prevalence. Medical treatment may reduce the physical and mental discomfort, while it has not been able to improve the possibilities for retaining or return patients with LBP to work.

This is an occupational intervention study for patients with LBP and physically demanding work, who are at risk of drop out of labour; a randomized controlled trial designed to test the effectiveness of an early intervention for retaining subjects with LBP attached to the labour marked. A work place modification intervention combined with moderate physical activity is given in the intervention group additional to LBP treatments according to best practice recommendations for general practice.

The study population consists of patients in self-reported physically demanding, who are sick listed or at risk of sick leave due to LBP. Outcome will continually be collected during the intervention as well as 6 and additionally at 12 months follow up.

The primary aim is to evaluate if an occupational intervention with focus on early workplace orientated counselling and work place intervention can retain subjects with physically demanding work and LBP in gainful employment to prevent/reduce the sick leave due to LBP.

The secondary aims are to identify prognostic factors of an occupational intervention using the baseline and follow-up participant-rated outcomes: pain, physically function, generic health status, fear avoidance behaviours, job satisfaction, work-ability, satisfaction with intervention, clinical examination and MRI findings. Among these variables, we also aim to identify subjects, who will benefit from such an occupational intervention, and the subjects, who already have a good prognosis and therefore have no need for a larger scale intervention.

Condition	Intervention	Phase
Low Back Pain	Behavioral: Occupational intervention<br>Usual care	N/A

Study Type: Interventional
Study Design: Treatment, Parallel Assignment, Single Blind (Outcomes Assessor), Randomized, Efficacy Study
Official Title: Retention in Physically Demanding Jobs With Low Back Pain: A Randomised Controlled Trial (GoBack)

Further study details as provided by Bjarke Brandt Hansen, Frederiksberg University Hospital:

Primary Outcome Measure:

- Accumulated duration of self-assessed sick leave due to LBP [Time Frame: 6 months] [Designated as safety issue: No]

Secondary Outcome Measures:

- Changes in pain level [Time Frame: 6 months] [Designated as safety issue: No]
  Pain will be evaluated by using the 13 items Pain-Detect questionnaire, which includes measurements of LBP on an ordinal, 11-point numerical rating scale (NRS: 0 = no LBP; 10 = worst LBP possible).
- Changes in Fear Avoidance Beliefs scores [Time Frame: 6 months] [Designated as safety issue: No]
  Both Fear Avoidance Beliefs Work Subscale and Physical Activity subscale (questionnaires) will be measured.
- Change in Disability [Time Frame: 6 months] [Designated as safety issue: No]
  The 24-item Roland Morris Disability Questionnaire will measure participant-rated LBP disability.
- Satisfaction with the intervention [Time Frame: 6 months] [Designated as safety issue: No]
  Measured on an 11-point NRS with the anchors “not at all satisfied” to “extremely satisfied”.

Other Pre-specified Outcome Measures:

- Accumulated duration of self-assessed sick leave due to LBP [Time Frame: 12 months] [Designated as safety issue: No]
- MRI findings [Time Frame: Baseline] [Designated as safety issue: No]
  Degree of degenerative MRI findings in the lumbar spine predicts outcome (sick leave)
- Predictors of outcome [Time Frame: Baseline] [Designated as safety issue: No]
  Baseline questionnaires scores (Pain categorization (PainDETECT®), Numeric Pain Rating Scale (NPRS), Fear Avoidance Beliefs, 36-item short-form health-survey (SF36) and Roland Morris Disability) are associated with outcome (sick leave).

Estimated Enrollment: 300
Study Start Date: January 2014
Estimated Study Completion Date: December 2017
Estimated Primary Completion Date: June 2016

Arms	Assigned Interventions
Experimental: Occupational intervention<br>Early coordinated occupational intervention. Supervision in physically activities by a physiotherapist.	Behavioral: Occupational intervention<br>Early coordinated occupational intervention and supervision in physically activities by a physiotherapist.
Active Comparator: Usual care<br>Intervention from the patient's general physician.	Usual care<br>Intervention from physiotherapist, chiropractor, rheumatologist coordinated by the patient's general physician

Eligibility

Ages Eligible for Study: 18 Years to 65 Years
Genders Eligible for Study: Both

Inclusion Criteria:

- Working age adults 18-65
- Low back pain (current episode of 2-4 weeks)
- Self-reported physically demanding work
- Sick-listed or at risk
- Speak, read and understand Danish
- Accept workplace visit by the occupational physicians
- Be in gainful employment for at last 30 hours/week

Exclusion Criteria:

- Severe somatic or psychiatric comorbidity
- Pregnancy
- Cancer or metastatic disease
- LBP treatment or referral to outside providers (e.g. back-surgery)
- Contraindications for having a MRI.

Contacts and Locations

Contacts

Ann Kryger, MD, PhD +45 35 31 60 75 ann.isabel.kryger@regionh.dk
Bjarke Hansen, MD +45 35 16 41 82 bjarke.hansen.01@regionh.dk

Locations

Denmark

The Parker Institute, Department of Rheumatology, Copenhagen University Hospital, Bispebjerg and Frederiksberg Hospital

Copenhagen, Frederiksberg, Denmark, 2000 F

Contact: Bjarke Hansen, MD +45 3516 41 82 bjake.hansen.01@regionh.dk

Contact: Ann Kryger, MD PhD +45 35 31 60 75 ann.isabel.kryger@regionh.dk

Sub-Investigator: Bjarke Hansen, MD

Sub-Investigator: Henning Bliddal, MD, DMSic

Principal Investigator: Ann Kryger, MD PhD

Sub-Investigator: Lilli Kirkeskov, MD

Sub-Investigator: Ellen Pedersen, MD

Sub-Investigator: John Bang, MD

More Information

Department of Occupational and Environmental Medicine, Copenhagen University, Hospital Bispebjerg and Frederiksberg

http://www.bispebjerghospital.dk/menu/Afdelinger/Kliniske+afdelinger/Arbejds+og+Miljoemedicinsk+...

The Parker Institute, Department of Rheumatology, Copenhagen University Hospital, Bispebjerg and Frederiksberg Hospital

http://www.parkerinst.dk/

Publications:

Jensen C, Jensen OK, Nielsen CV. Sustainability of return to work in sick-listed employees with low-back pain. Two-year follow-up in a randomized clinical trial comparing multidisciplinary and brief intervention. BMC Musculoskelet Disord. 2012 Aug 25;13:156. doi: 10.1186/1471-2474-13-156. PubMed

Responsible Party: Bjarke Brandt Hansen, MD, Frederiksberg University Hospital

Study ID Numbers: GoBack-001

Health Authority: Denmark: Danish Dataprotection Agency

Denmark: The Regional Committee on Biomedical Research Ethics
