# Supplementary material for: Early occupational intervention for people with low back pain in physically demanding jobs: A randomized clinical trial
Source: PLoS Med. 2019 Aug 16;16(8):e1002898. doi: 10.1371/journal.pmed.1002898 (PMC6697316; doi:10.1371/journal.pmed.1002898)
Supplement: S1 Alternative Language Abstract — (PDF) [file pmed.1002898.s008.pdf]

Danish summary:

Tidlig arbejdsmedicinsk indsats til personer med lænderygsmerter og fysisk krævende jobs: Et randomiseret klinisk forsøg

Baggrund

Ved en tidlig arbejdsmedicinsk indsats forsøger man at reducere sygefravær, men der er imidlertid begrænset viden om den potentielle adderende effekt, hvis man forudgående har givet patienterne en grundig ikke-kirurgisk udredning i reumatologisk regi. Formålet med GoBack-studiet var at undersøge en tidlig 3 måneders fokuseret arbejdsmedicinsk indsats forudgået af en MR skanning af ryggen og en grundig reumatologisk undersøgelse reducerede personernes sygefravær. Den komplekse arbejdsmedicinske indsats, indeholdt samtaler med en arbejdsmediciner, arbejdspladsbesøg og samtaler med en fysioterapeut, hvis formål var at øge det ugentlige fysiske aktivitets niveau. Alle deltagere fik forudgående en udredning i reumatologisk regi og en MR skanning.

Metoder og resultater

GOBACK-studiet er et åbent, randomiseret klinisk forsøg, som blev gennemført fra marts 2014 til december 2015 i samarbejde mellem arbejdsmedicinsk afdeling, Bispebjerg Hospital og læger fra Parker Instituttet, Frederiksberg Hospital. 305 deltagere (99 kvinder) med lænderygsmerter og fysisk krævende arbejde blev randomiseret til en kompleks arbejdsmedicinsk indsats og vejledning fra en fysioterapeut (indsatsgruppen; n=153) eller ingen ekstra indsats (kontrolgruppe; n=152). Indsatsen blev foretaget som tillæg til en ikke-kirurgisk udredning med MR i reumatologisk regi. Den forudgående ikke-kirurgiske udredning indeholdt også opfordringer til patienten om at forblive aktiv og fortsætte med at arbejde. Primær outcome var akkumuleret sygefravær på grund af lænderygsmerter over 6 måneder. Sekundære outcomes var: ændringer i neuropatisk smerte: (painDETECT- Questionnaire [PDQ]), smerte: 0-10 Numerisk Rating Scale (NRS), frygt for sygdomsforværring: Fear Avoidance Beliefs Questionnaire FABQ, funktionsbegrænsning: Roland Morris Disability Questionnaire (RMDQ), livskvalitet: Short Form Health Survey (SF- 36) for fysisk og psykisk livskvalitet (HRQoL) og selvvurderet evne til at fortsætte med at arbejde (Range: 0-10).

En ITT-analyse af sygefravær over 6 måneder viste ingen signifikant forskel mellem grupperne (gennemsnitlig forskel: 3,50 dage [95% CI, -5,08 til 12,07], P = 0,42). Begge grupper havde signifikante forbedringer i gennemsnitlige smerte (NRS), funktionsbegrænsning (RMDQ), frygt for sygdomsforværring ved fysiske aktiviteter og arbejde (FABQ), samt fysisk livskvalitet HRQoL (SF-36-PCS). Der var ingen signifikante forskelle mellem grupperne på alle sekundære outcomes og ingen statistisk forbedring over de 6 måneder i forhold til neuropatisk smerte (PDQ-score), mental livskvalitet HRQoL (SF-36-MCS) og selvvurderet evne til at blive i arbejde. Fire deltagere kunne ikke gennemføre MR skanningen og en måtte stoppe studiets forsøgsbehandling grundet forværring af lænderygsmerterne.

Arbejdspladsbesøg kan være et vigtigt element i den arbejdsmedicinsk indsats. En Per-protokol analyse, der omfattede de 40 deltagere, der modtog arbejdspladsbesøg viste ikke yderligere reduktion i sygefravær over de 6 måneder (gennemsnitlig forskel mellem grupperne: -0,43 dage [95% Cl, -12,8 til 11,94], P = 0,945). Et begrænset sygefravær i begge grupper, samt anvendelse af MR til alle deltagere var studiet størst svaghed.

Konklusion

Når man giver en grundig ikke-kirurgisk udredning i reumatologisk regi med MR, havde personer i fysisk krævende arbejde med lænderygsmerter ikke fordel af en kompleks arbejdsmedicinsk indsats. Den arbejdsmedicinske indsats for patienter med lænderygsmerter sigter mod at reducere bio-psyko-sociale forhindringer for at forblive i arbejde (f.eks. frygt for at fysisk aktivitet og frygt for at arbejdet forværrer ens sygdom), samt reducere arbejdsrelaterede belastninger på arbejdspladsen. Dette studie indikerer, at disse bio-psyko-sociale faktorer kan håndteres ved en grundig ikke-kirurgisk indsats efter vejledning fra arbejdsmedicinske læger.

GoBack-studiet var støttet økonomisk af Danish Working Environment Research Fund and OAK foundation

(Clinical Trials.gov: NCT02015572)
